# A multispectral imaging approach integrated into the study of Late Antique textiles from Egypt

**DOI:** 10.1371/journal.pone.0204699

**Published:** 2018-10-04

**Authors:** Joanne Dyer, Diego Tamburini, Elisabeth R. O’Connell, Anna Harrison

**Affiliations:** 1 Department of Scientific Research, The British Museum, London, United Kingdom; 2 Department of Ancient Egypt and Sudan, The British Museum, London, United Kingdom; 3 Department of Conservation, The British Museum, London, United Kingdom; University at Buffalo - The State University of New York, UNITED STATES

## Abstract

This work explores the use of multispectral imaging (MSI) techniques applied to the investigation of Late Antique (c. 250–800 AD) textiles found in Egypt. Although the use of these techniques is well-established in the study of polychrome surfaces, they have only been sparingly and often unsystematically applied to the investigation of textiles. The aim of this work is therefore to bridge this gap by showing how this non-invasive, relatively inexpensive and portable methodology can be used to map the photoluminescence and reflective characteristics of textiles under different wavelengths of light, and to provide qualitative and holistic insights into the chemical nature of the materials that compose them. Standardised acquisition and post-processing methods were applied to produce visible-reflected (VIS), ultraviolet-induced visible luminescence (UVL), infrared-reflected (IRR), infrared-reflected false colour (IRRFC), ultraviolet-reflected (UVR) and ultraviolet-reflected false colour (UVRFC) images that provided preliminary indications of the colourants used and their spatial distribution. This proved to be an important aid in planning more targeted and effective sampling strategies and facilitated comparisons between objects. Visible-induced visible luminescence (VIVL) and multiband-reflected (MBR) imaging were also explored for the first time with application to textiles, demonstrating their potential in mapping red and blue colourants respectively. The physical properties observed from all of these images were then related to the more detailed information provided by complementary non-invasive techniques, such as fibre optic reflectance spectroscopy (FORS), and micro-invasive approaches, such as high-performance liquid chromatography mass spectrometry (HPLC-MS). Guidelines towards the interpretation of complex MSI images and a discussion of the potential and limitations of relating multispectral data to chemical properties are presented. An important result of this work is the delineation of a protocol, which combines optical microscopy (OM), MSI, FORS and HPLC-MS and shows a high degree of potential, not only for the investigation of Late Antique textiles but for textiles in museum and historic collections generally.

## Introduction

Multispectral imaging (MSI) techniques are a set of broadband photographic methods specifically adapted to map the photoluminescence and reflective characteristics of surfaces under different wavelengths of light. When conducted using standardised acquisition and post-processing methods, information about the chemical nature of the materials present and their spatial distribution can be obtained. This can also provide guidance in planning more targeted and effective sampling strategies and facilitate comparisons between objects [1].

Although the use of MSI techniques is well-established in the study of polychrome surfaces [2–5], these have only been used sparingly in the investigation of historical or archaeological textiles. Coremans’ use of near infrared (NIR) photography in 1938, to document tapestry restorations [6], was an early adoption of employing wavelengths of light outside of the capabilities of the human eye in the investigation of textiles. However, only recently have more scientific applications appeared in the literature. Of these, Borrego and Vega’s use of multispectral techniques, particularly ultraviolet-induced visible luminescence (UVL) to differentiate between linen and wool fibres in Late Antique textiles from the Soler Vilabella collection at the Museum of Monserrat (Barcelona, Spain), is of note [7]. The use of multispectral techniques, such as UVL and infrared-reflected (IRR) imaging were also effectively employed by Haldane *et al*. to document stains on a Late Antique Egyptian tunic from the collections of the Victoria and Albert Museum [8].

Despite these important applications in both the differentiation of materials and the documentation of textiles (interestingly both on Late Antique textile collections) little has been done [9] specifically on the use of these techniques to investigate the photoluminescence and reflective properties of the dyes and colourants. Often faded, the (once) highly-coloured appearance of many historic or archaeological textiles is a defining aspect and imperative to understanding their provenance, their interpretation and their placement in a socio-economic context.

Fewer attempts still [10] have been made to consider a systematic approach towards both the acquisition and post-processing of these images or to establish protocols to ensure that problems of reproducibility and comparability are addressed. As a result, a series of difficulties in the interpretation of these images is encountered and the valuable information obtained becomes overly subjective and often meaningless.

It is clear from this overview that a set of techniques specifically adapted to consider the coloured aspect of textiles, as well as other characteristics of their materiality, is needed. Standardised acquisition and post-processing methods, which have been scientifically optimised, are required to afford preliminary indications of the colourants present and facilitate comparisons between objects. In addition, a complete set of images should include both reflectance and luminescence images, that consider not only emitting materials but also their reflectance and absorbance properties, which are often characteristic and diagnostic for their identification.

To obtain such an optimised protocol, the colour-calibration of visible images (VIS) is fundamental, as it improves comparability between images taken at different times and/or locations. The creation of this high-resolution reference image, over which the other images can be registered, is important for the effective comparison and interpretation of the image set and the creation of balanced false colour images. UV-induced visible luminescence imaging (UVL) can then be used to map the visible luminescence emitted from the dyed yarns and begin to relate this to their chemical sources. As discussed, infrared-reflected imaging (IRR) is useful for the documentation of staining or past restorations but can also effectively be used in the visualisation of metal threads [10]. Perhaps most significantly, infrared-reflected false colour (IRRFC) images can be created from the IRR and VIS images. IRRFC images can allow preliminary identification of colourants due to their characteristic appearance in false colour, as determined by their combined absorbance or reflectance properties in the visible and infrared ranges. Although rarely used in the context of textile analysis, UV-reflected imaging (UVR) can be useful for the detection of patterns, staining, surface coatings and, similarly to the IRR images, are also useful for revealing previously unseen areas of restoration and creating UV-reflected false colour (UVRFC) images.

Additionally, two novel MSI methods have been explored for the first time, which are believed to be of particular value for the study of textiles. The first of these is a luminescence technique analogous to UVL imaging, but which uses visible light, specifically blue light in this instance, as the excitation source. Visible-induced visible luminescence imaging (VIVL) has recently been applied to the study of polychrome sculpture with, among others, the notable advantage that it dispenses with the use for UV sources, thus making it safer for both user and object [2]. In its application to textiles the VIVL technique has a further advantage over UVL imaging, in that the longer excitation wavelength used avoids the absorption maximum of most fibres [11], thus suppressing background luminescence from these and minimising the distracting and colour-modifying effects which this can cause. The second method is a mapping technique which specifically targets the reflectance of colourants, such as indigo, by acquiring reflectance images in multiple bands and subtracting these to produce an image which highlights the spatial distribution of the indigo-containing areas. The technique, termed multiband-reflected (MBR) imaging, is described in relation to archaeological Andean painted textiles by Webb *et al*. but has not hitherto been published with applications to dyed woven textiles [12].

Once acquired and processed, the interpretation of complex MSI images is often a source of difficulty for those who are unfamiliar or in cases where little previous reliable data exists. A further aim of this work is to provide guidelines which will aid in the systematic interpretation of these images, relating multispectral data to chemical properties, whilst discussing the advantages and limitations of this approach.

It is always important to acknowledge that, depending on the detail of information needed, the photophysical properties observed and the preliminary chemical information obtained from MSI techniques requires confirmation and/or amplification using complementary techniques. Fibre optic reflectance spectroscopy (FORS) remains a non-invasive approach and has shown its potential in the identification of dyes [13–15]. When the removal of samples is feasible and permissible, high performance liquid chromatography mass spectrometry (HPLC-MS) is considered the state-of-the-art technique for the investigation of dyes, as it provides information at the molecular level and can allow for the distinction of the botanical/animal sources of colourants [16–22]. As a micro-invasive approach, the amount of sample needed is usually in the range of 50–100 μg (2–3 mm of fibres).

Aside from exploring the use of MSI techniques for the investigation of textiles and aiding in the interpretation of the images produced, this work illustrates how these techniques can be integrated into a multi-analytical protocol in combination with FORS and HPLC-MS (on selected samples) to provide a better understanding of historical/archaeological textiles. For the samples selected for HPLC-MS analysis, the protocol proposed additionally includes investigation by optical microscopy (OM) and the analysis of sub-samples (one-two fibres) by scanning electron microscopy with energy dispersive X-ray spectroscopy (SEM-EDX) for mordant identification.

### Research context and aims

The protocol is here presented with specific application to Late Antique textiles from Egypt in the British Museum collection. Until recently, the origin and date of such textiles were largely determined by style and iconography. Increasingly, the application of scientific techniques is slowly over-turning long-held assumptions concerning chronology, the sources of materials and availability of dyestuffs, and providing new insights into textile production, dyeing practices, trade and the economy.

For this research four objects were selected; one is from the rubbish dumps of the city of Antinoupolis [23] and three are from the Monastery of Apa Thomas at Wadi Sarga, both in Middle Egypt [24,25], as there was a strong curatorial interest in the comparison of textiles to assess differences in practice as associated with urban versus more rural, monastic contexts. A particular emphasis was placed on the use of dyes, as, in addition to informing on the skills and ingenuity of dyers in Late Antique Egypt, dye analysis can potentially provide information on trading of dyes and provenance of the raw materials. The possibility to identify such dyes, in cases where these may now have faded, also yields the opportunity to better appreciate and/or reconstruct their original appearance. In cases where the dyes are better preserved, these studies also provide the information required with which to design and implement more responsible strategies for the display of such vulnerable textiles.

These motivations formed the context and aims of this research project, and although the increased knowledge of the materials and techniques used to create these pieces will be briefly discussed, the main focus of this paper is the important opportunity that this research provided to explore multi-spectral imaging as a tool to begin to answer these questions and to evaluate the advantages and limitations of the method when integrated into an analytical protocol.

## Materials and methods

### Objects and samples

**Fig 1:**
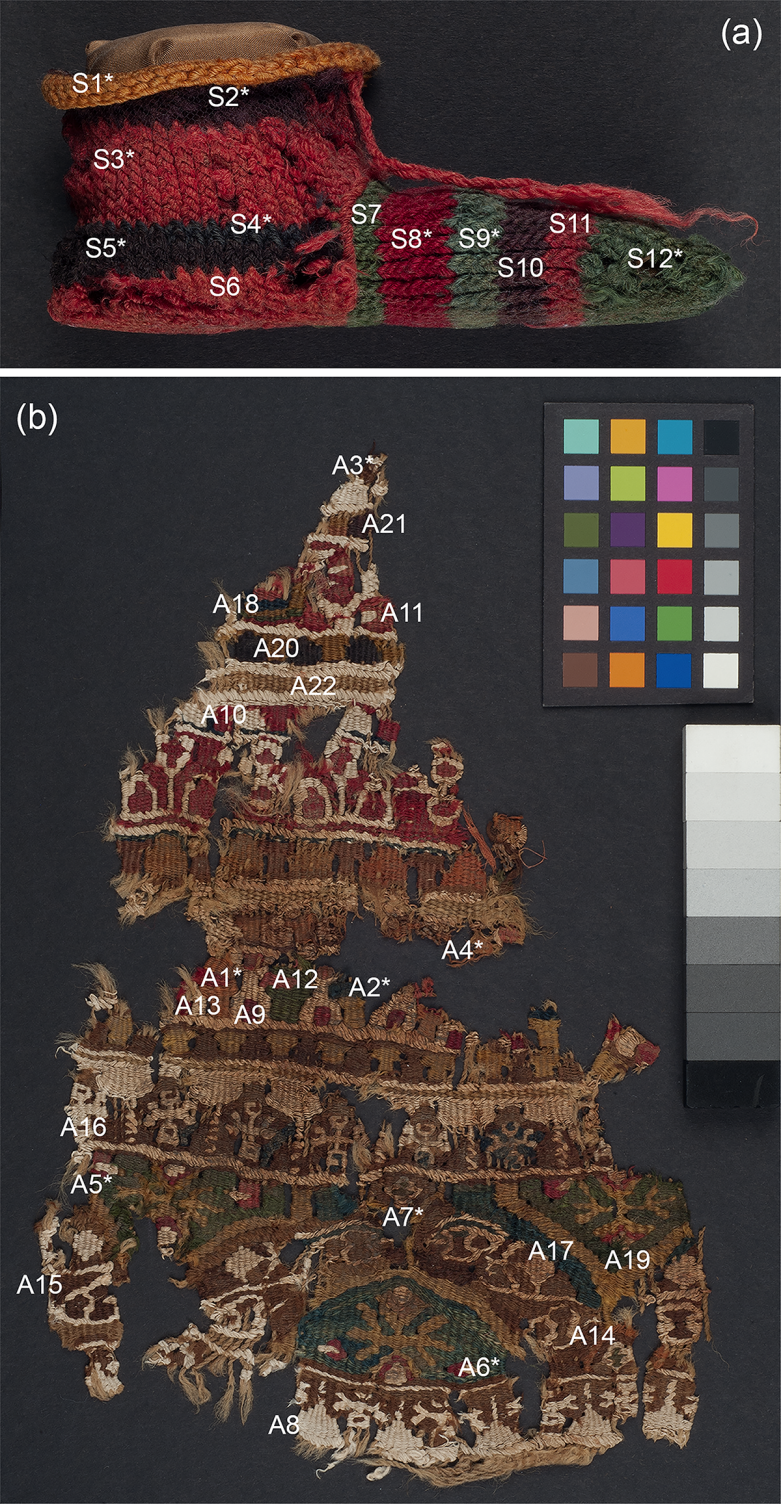
(a) A child’s stripy sock from Antinoupolis (EA53913) and (b) textile fragment A from Wadi Sarga (EA72555).

**Fig 2:**
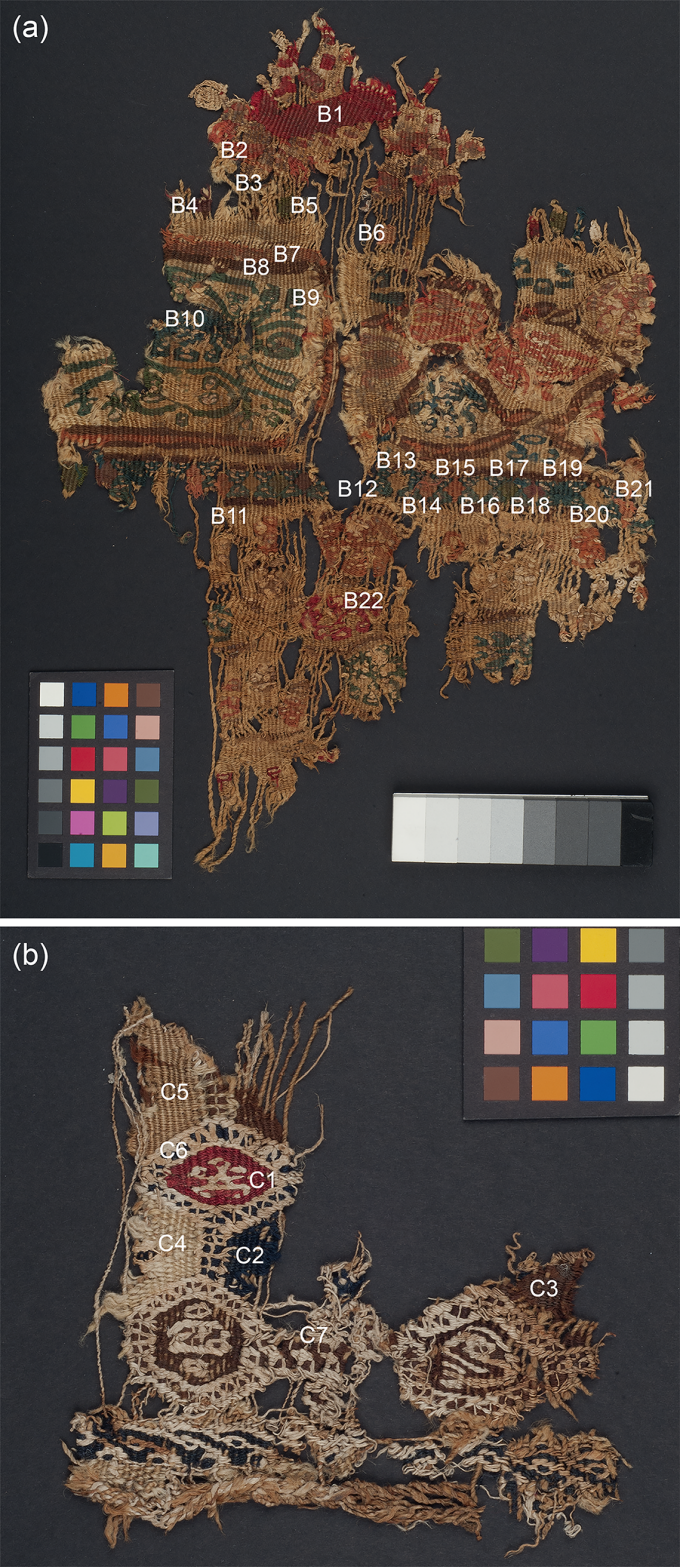
(a) Textile fragment B (EA72551) and (b) textile fragment C (EA72553) from Wadi Sarga.

Four objects from the British Museum collection were selected for their historical/archaeological importance. A child’s stripy sock from Antinoupolis (registration number EA53913; Fig 1A) and a fragment of a furnishing textile from Wadi Sarga (henceforth known as textile fragment A; registration number EA72555; Fig 1B) were studied with all techniques. Two additional fragments of furnishing textiles from Wadi Sarga (henceforth known as textile fragments B and C, registration number EA72551; Fig 2A, and registration number EA72553; Fig 2B, respectively) were analysed by MSI and FORS only. These textiles are held in the Department of Ancient Egypt and Sudan at the British Museum (London, UK).

#### A child’s stripy sock

This sock was for the left foot of a child with separation between the big toe and four other toes worked in 6–7 colours of wool yarn (several S-spun strands, Z-plied) in a single needle looping technique sometimes called nålbinding and worked from the toe upwards. Each toe is made separately from dark green wool (10 rows). The two toes are then joined and worked in bands of the following colours: orange (4 rows), purple (4 rows), bluish-green (4 rows), dark red (6 rows), green (2 rows). The sole of the heel is then worked. The heel section is worked in bands of orange (3 rows), purple (3 rows), dark blue (2 rows), orange (8 rows), purple (4 rows), yellow (4 rows). A welt across the instep marks where the loops are worked in the round. The top edge is continuous and curls over; a loose thread of red wool forms part of a tie or tassel at the centre front [23]. The sock was radiocarbon-dated to 3^rd^-4^th^ century AD [26].

To aid in the discussion of both the multispectral imaging and the analytical results, the sock was divided into twelve areas, in accordance with each of the coloured stripes observed, and labelled (S1—S12), as recorded in Fig 1A. FORS measurements were performed at each of these areas. In addition, eight micro-samples (marked with an asterisk on Fig 1A) were taken from representative areas.

#### Textile fragment A

This fragment of a furnishing textile was made of wool and linen. The design is organised in bands with, from the bottom, a semi-circle of stylised plants in white on brown, enclosing, on a blue ground, a green cruciform plant motif with red and white flowers; in the spandrels, similar motifs on dark green; a narrow band in blue, white and brown, the repeating motif, again, plant-like; a poorly preserved band, multi-coloured on a white ground; a band with repeating motifs in white on a red ground; another, wider, multi-coloured band [24,25]. The textile was radiocarbon-dated to 660–770 AD [27].

To similarly facilitate discussion of both the multispectral imaging and the analytical results, areas representative of the coloured yarns which make up the textile fragment were selected and labelled (A1 –A23), as recorded in Fig 1B. FORS measurements were performed at each of these areas. In addition, eight micro-samples (marked with an asterisk on Fig 1B) were taken from representative areas where sampling was possible.

#### Textile fragment B

This woven wool textile also shows the presence of some linen. The design is organised in bands. The main band has borders of multi-coloured diamonds or squares on a blue ground and pink and brown stripes; on the left, are curling plant stems in green with an unrecognisable motif at centre and, on the right, are almond-shaped compartments framed in pink and brown and enclosing unrecognisable motifs in pink and blue. Above and below, poorly preserved, is a design predominantly in red, pink, blue and green. The textile is stylistically dated to 7^th^-8^th^ c. AD [24,25].

#### Textile fragment C

This textile shows red and dark blue wool decorations and various natural (undyed) shades of wool. The design consists of small lozenges or hexagons containing stylised motifs joined by bars to form a grid [24,25]. It was radiocarbon-dated to 555–650 AD [27].

As with the previous examples, areas representative of the coloured yarns, which make up the textile fragments, were selected. These areas, labelled B1 –B22 for textile fragment B and C1—C7 for textile fragment C, are recorded in Fig 2. FORS measurements were performed at each of these areas.

### 2.2. Analytical methods

The multi-analytical protocol discussed was applied in order to provide a better understanding of the archaeological textiles under investigation. The main aim was to collect as much information as possible non-invasively, to minimise the number of samples to be taken for destructive analysis.

**Fig 3:**
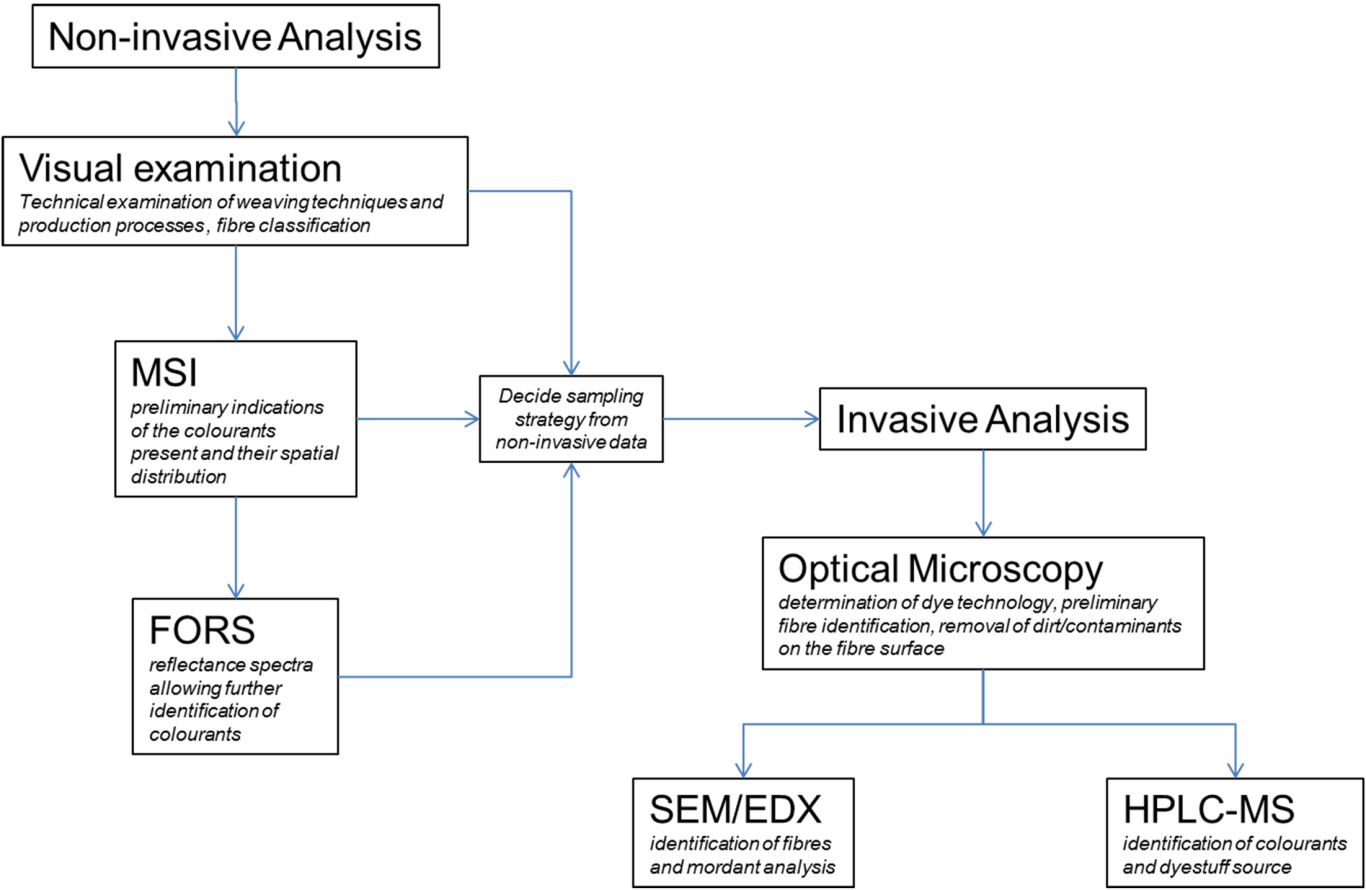
Protocol applied for the examination of archaeological textiles integrating MSI techniques with FORS, OM, SEM-EDX and HPLC-MS.

Non-invasive techniques, including a preliminary visual examination of the textiles (weaving technique, production process and fibre classification), followed by multispectral imaging (MSI) and fibre optic reflectance spectroscopy (FORS) constituted the first stages of this protocol as described in Fig 3.

At this stage some of the colourants could already be identified, but where further analyses were required, the information obtained by MSI and FORS enabled a strategic sampling campaign to be performed. A low number of samples were taken based on similarities between coloured areas observed in the MSI image sets and the need for further investigation by the invasive methods described in the second stage of the protocol (Fig 3).

The samples were observed using OM, which is useful to differentiate between yarns dyed with a single colour or a mixture of colours at the fleece or yarn stage; yarns composed of different colours that are dyed separately (at the fleece or yarn stage) and are then spun or twisted together to obtain a mixed colour; and double-dyed yarns where sequential dye baths are applied to the same fleeces or yarns.

One or two fibres were then separated from each sample for SEM-EDX analysis of mordants. The residual samples underwent extraction prior to HPLC-MS analysis.

#### Multispectral imaging techniques (MSI)

All images were taken using a modified Canon 40D camera body. The modification consists of the removal of the inbuilt UV-IR blocking filter, to exploit the full sensitivity of the CMOS sensor (c. 300–1000 nm). The lens used is a Canon EF 50mm f/1.8II. The camera is operated in fully manual mode. A reference grey scale, comprising a set of Lambertian black, grey and white tiles, is placed in the same plane as the object under investigation. These Spectralon references have uniform reflectance properties across the ultraviolet, visible and infrared spectral ranges under investigation and show no luminescence properties [28].

In each case the object is illuminated by two radiation sources symmetrically positioned at approximately 45° with respect to the focal axis of the camera and at about the same height. A filter, or combination of filters, is placed in front of the camera lens in order to select the wavelength range of interest. The combinations of radiation sources and filter(s) used for each MSI technique are summarised in Table 1.

**Table 1:** Summary of the combination of radiation sources and filter(s) used for each of the multispectral imaging techniques considered.

MSI Technique	Radiation Sources	Filter(s) in front of camera	Range investigated	Refs
**Visible-reflected imaging (VIS)**	2 x Classic Elinchrom 500 Xenon flashlights, each equipped with a softbox (diffuser)	IDAS-UIBAR interference UV-IR blocking bandpass filter (c. 380–700 nm)	c.380–700 nm	[1]
**Ultraviolet-induced visible luminescence imaging (UVL)**	2 x Wood’s radiation sources (365 nm) filtered with a Schott DUG11 interference bandpass filter (280–400 nm)	Schott KV418 cut-on filter (50% transmission at c. 418 nm) + IDAS-UIBAR bandpass filter (c. 380–700 nm)	c. 420–700 nm	[1]
**Infrared-reflected imaging (IRR)**	2 x Classic Elinchrom 500 Xenon flashlights, each equipped with a softbox (diffuser)	Schott RG830 cut-on filter (50% transmittance at c. 830 nm)	c. 800–1100 nm	[1]
**Ultraviolet-reflected imaging (UVR)**	2 x Wood’s radiation sources (365 nm) filtered with a Schott DUG11 interference bandpass filter (280–400 nm)	Schott DUG11 interference bandpass filter (280–400 nm)	c. 350–400 nm	[1]
**Visible-induced visible luminescence imaging (VIVL)**	2 x high power LED (red, green and blue) light sources (Eurolite LED PAR56 RGB spots 20W, 151 LEDs, beam angle 21^o^). Blue LEDs (λ_max_ = 465 nm)	IDAS-UIBAR bandpass filter (400–700 nm) + Tiffen Orange 21 filter (50% transmission at 550 nm)	c. 540–700 nm	[2]
**Multiband-reflected imaging (MBR)**	2 x Classic Elinchrom 500 Xenon flashlights, each equipped with a softbox (diffuser)	MidOpt BP 660 dark red bandpass filter (c. 640-680nm) then MidOpt BP735 infrared bandpass filter (715-780nm)	N/A	[12]

All images are acquired as RAW images and transformed into 3888 × 2592 pixel resolution images in 16-bit TIF (tagged image file) format, and by turning off all enhancements (e.g. recovery, fill light, blacks, contrast, brightness, clarity, vibrance, saturation, as well as setting the tone-curve to linear). This procedure can be carried out using the camera software or external programs such as Adobe Photoshop. For further details on the conversion of images from RAW, see the manual on multispectral imaging techniques [1].

Post-processing procedures for the standardisation and calibration of the VIS, IRR, UVL and UVR images and the creation of IRRFC and UVRFC images are then carried out using “BM_workspace”, a plug-in for Nip2, the open-source graphical user-interface of VIPS, a free image processing software [29]. For details on how to download BM_workspace and the Nip2 software, as well as descriptions, workflows and data requirements for the post-processing of these images, see the manual on multispectral imaging techniques [1].

Post-processing procedures for the VIVL and MBR images are not currently supported by the BM_workspace. However a post-processing protocol for VIVL images was described in Dyer et al. [2]. Image subtraction of the MBR images was undertaken in Adobe Photoshop to provide the final image [12].

#### Fibre optic reflectance spectroscopy (FORS)

Fibre optic reflectance spectra were recorded with an Avantes (Apeldoorn, The Netherlands) AvaSpec-ULS2048XL-USB2 spectrophotometer equipped with an AvaLight-HAL-S-IND tungsten halogen light source. The detector and light source were connected with a fibre optic bundle to an FCR-7UV200-2-1.5 × 100 probe. In this configuration, light was sent and retrieved by the bundle set at approximately 45° from the surface normal, thus excluding specular reflectance. The spectral range of the detector was 200–1160 nm; nevertheless, due to poor blank correction on both the extremes of the range, only the range between 350 and 900 nm was considered; as per the features of the monochromator (slit width 50 μm, grating of UA type with 300 lines/mm) and of the detector (2048 pixels), the best spectra resolution was 2.4 nm calculated as full width at half maximum (FWHM). Spectra were referenced against the WS-2 reference tile provided by Avantes. The diameter of the investigated area on the sample was approximately 1 mm, obtained by setting the distance between probe and sample at 1 mm. The instrumental parameters were as follows: 100 ms integration time, 5 scans for a total acquisition time of 0.5 s for each spectrum. The whole system was managed by the software AvaSoft 8 for Windows.

#### Optical microscopy (OM)

The samples were placed on a glass slide and photographed under a Leica MS APO microscope with reflected light and a magnification of x80.

#### Scanning electron microscopy–energy dispersive X-ray spectroscopy (SEM-EDX)

Each sample was placed uncoated on an adhesive carbon disc mounted onto an aluminium SEM stub; no other sample preparation was undertaken. Examination of the samples was carried out in the variable pressure VP SEM (Hitachi S-3700N) using the backscatter electron (BSE) detector mostly at 16 kV. Magnifications ranged from x20 to x750. The working distance was 10 mm. The SEM chamber was only partially evacuated (mostly 40 Pa).

The EDX spectra were collected using an Oxford Instruments INCA EDX spectrometer with a 0–20 KeV spectral range, 150 seconds live time number of channels 2048. AZtecEnergy analysis software (Oxford Instruments) was used to process the data.

#### High pressure liquid chromatography–diode array detector–electrospray ionisation–quadrupole–time of flight (HPLC-DAD-ESI-Q-ToF)

A double-extraction combined procedure was adopted to solubilise the dye molecules from the fibres. Approximately 0.1 mg (2–3 mm) of sample were admixed with 200 μL DMSO and heated at 80°C for 10 minutes. After centrifugation, the supernatant was transferred into another vial. The residue was admixed with 200 μL of methanol/acetone/water/0.5M oxalic acid 30:30:40:1 (v/v/v/v) and at 80°C for 15 minutes. The solution was evaporated under N_2_ and reconstituted using 200 μL of 1:1 MeOH/H_2_O (v/v). The DMSO extract was combined with the oxalic acid extract and the solution was centrifuged for 10 minutes. The supernatant was transferred to a fresh 250 μL insert and 10–30 μL of the solution were injected into the HPLC system. The extraction procedure was optimised in the framework of the CHARISMA project (2009–2014) funded by the European Union FP 7 Research Infrastructures programme (CHARISMA Grant Agreement no. 228330) and has proven its suitability for the analysis of organic colourants in both dyes [21,30] and pigments formulations [22,31].

Analyses were carried out using a 1260 Infinity HPLC (Agilent Technologies), coupled to a 1100 DAD detector (Hewlett-Packard) and a Quadrupole-Time of Flight tandem mass spectrometer 6530 Infinity Q-ToF detector (Agilent Technologies) by a Jet Stream ESI interface (Agilent Technologies).

The HPLC conditions were: Zorbax Extend-C18 column (2.1 mm × 50 mm, 1.8 μm particle size); 0.4 mL/min flow rate; 40°C column temperature. Separation was achieved using a gradient of water with 0.1% formic acid (eluent A) and acetonitrile (eluent B). The elution gradient was programmed as follows: initial conditions 95% A, followed by a linear gradient to 100% B in 10 min, held for 2 min. Re-equilibration time for each analysis was 10 min.

The ESI operating conditions were: drying gas (N_2_, purity >98%): 350°C and 10 L/min; capillary voltage 4.0 KV; nebulizer gas 40 psig; sheath gas (N_2_, purity >98%): 375°C and 11 L/min.

High resolution MS and MS/MS spectra were acquired in negative and positive modes in the range 100–1700 m/z. The fragmentor was kept at 150 V, nozzle voltage 1000 V, skimmer 65 V, octapole RF 750 V. For the MS/MS experiments, different voltages in the collision cell were tested for Collision Induced Dissociation (CID), in order to maximise the information obtained from the fragmentation. The collision gas was nitrogen (purity 99.999%). The data were collected by targeted MS/MS acquisition with an MS scan rate of 1.0 spectra/sec and an MS/MS scan rate of 1.0 spectra/sec. MassHunter Workstation Software was used to carry out mass spectrometer control, data acquisition, and data analysis.

The identification of the molecules in the samples was based on the comparison of the retention times and MS/MS spectra of reference molecules present in the BM database.

## Results and discussion

### Multispectral imaging

The multispectral images acquired from the child’s stripy sock and the furnishing textile fragments A—C are discussed in turn in the following sections. Systematic observation of the photophysical properties exhibited by each object and what these suggest about the chemical nature of the materials present are highlighted throughout. VIVL and MBR images were only recorded for the child’s stripy sock and textile fragment A, and the observations made from these images are discussed in a separate section.

#### Child’s stripy sock

**Fig 4:**
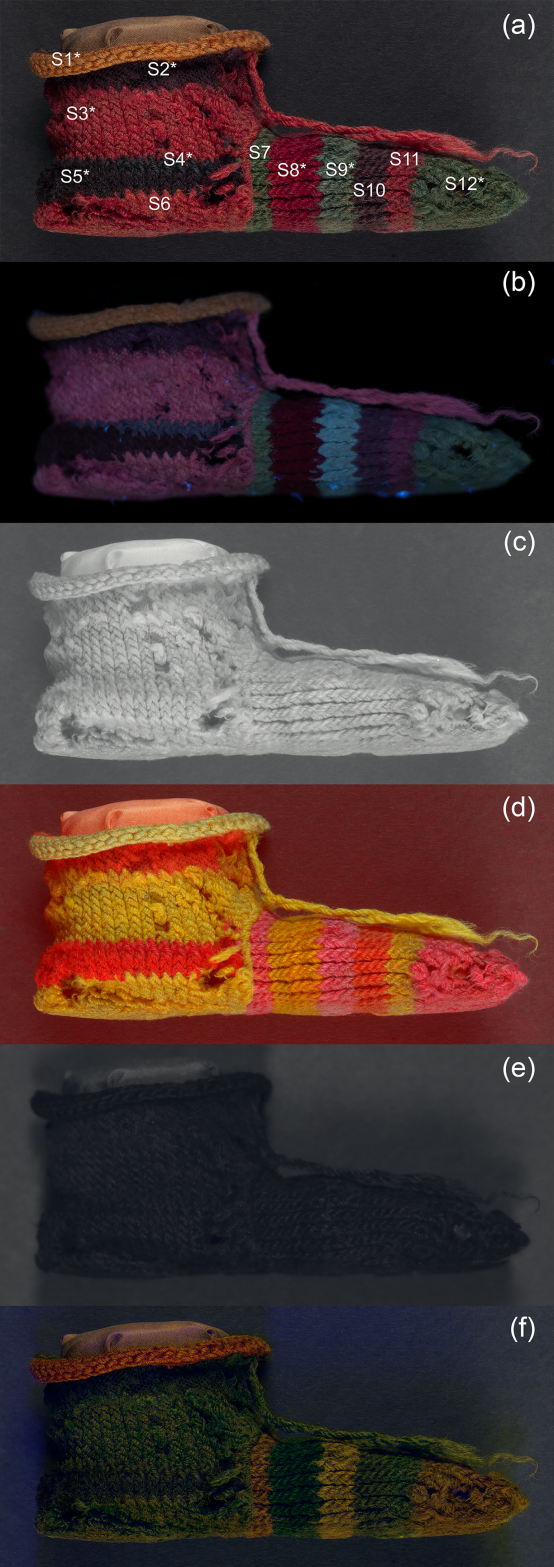
(a) Visible-reflected (VIS); (b) UV-induced visible luminescence (UVL); (c) Infrared-reflected (IRR); (d) Infrared-reflected false colour (IRRFC); (e) UV-reflected (UVR); (f) UV-reflected false colour (UVRFC) images of a child’s stripy sock (EA53913).

Fig 4 shows the set of multispectral images of the sock acquired and/or produced following post-processing. The main observations made from each image are detailed below.

#### UV-induced visible luminescence imaging (UVL)

The orange stripes, particularly S3 and S6, were observed to emit bright pink luminescence in the UVL image (Fig 4B). This behaviour is typically associated with red colourants such as madder [32,33], suggesting that this colourant may have been used alone or perhaps in a mixture with a yellow dye to produce this coloured yarn.

By contrast, stripe S8, which is a deeper red in the visible image, appears to absorb UV and is darker in the UVL image (Fig 4B). The difference in the behaviours of these areas of red colourants under UV irradiation was noteworthy.

A variation in the luminescence properties under UV irradiation for the green areas that appear similar in visible light was observed, with stripe S9 appearing much brighter and bluer in the UVL image (Fig 4B) compared to the weak green luminescence from stripes S7 and S12.

Differences in the luminescence properties of the dark purple stripe S5, and the visually similar stripes S2 and S10 were also observed. The former is quite UV-absorbing and largely appears dark in the UVL image, whereas the latter two emit a weak pinkish luminescence (Fig 4B), perhaps suggesting the presence of some madder in the dye composition.

Interestingly, a region of dull blue-grey luminescence is observed at the top of stripe S5 (Fig 4B), which is very different from the surrounding area. This difference in the UVL luminescence behaviour allowed this area to be correlated with an area of dark blue wool (stripe S4), which was hardly discernible in the visible range, revealing that the stripe is composed of two visually similar but differently coloured yarns.

#### Infrared-reflected imaging (IRR) and infrared-reflected false colour (IRRFC)

The materials which compose the sock are largely transparent to IR radiation, as suggested by the very light appearance of the IRR image (Fig 4C). However, the various colourants attenuate this transparency in various ways, which in turn affects the reflectance behaviour observed.

This difference in transparency/reflectance behaviour is evident from the IRRFC image (Fig 4D). Thus, the orange and deeper red stripes in the visible range (S3, S6, S8 and S11) appear bright yellow in the IRRFC image, with stripe S8 a slightly deeper hue (Fig 4D). The yellow stripe S1 appears a very pale yellow. The other stripes appear various shades of orange or pink. In IRRFC images, materials which absorb in the visible but not in the infrared range appear bright red [34]. Indigo exhibits this behaviour, but this transparency is often modified if it is present in mixture with other colourants. Mixtures with yellow dyes to produce green coloured materials often appear a salmon pink colour in the IRRFC image, whereas mixtures with reds, to give purple/brown coloured mixtures can appear bright orange: both are observed in the IRRFC image (Fig 4D), suggesting the presence of mixtures of indigo with yellow dyes (stripes S7, S9 and S12) and indigo with red dyes (stripes S2, S5 and S10), respectively, thus creating a map of indigo-containing areas.

#### UV-reflected imaging (UVR) and infrared-reflected false colour (UVRFC)

By contrast, the materials which make up the sock are mostly absorbing UV radiation, and as such, the UVR image appears very dark (Fig 4E). Although less apparent than for the IRRFC image, some differences in reflectance behaviour, resulting from different colourants present, are also evident from the UVRFC image. The green stripes (S7, S9 and S12) are particularly highlighted in this image, appearing a dull olive green (Fig 4F).

#### Textile fragments A, B and C

**Fig 5:**
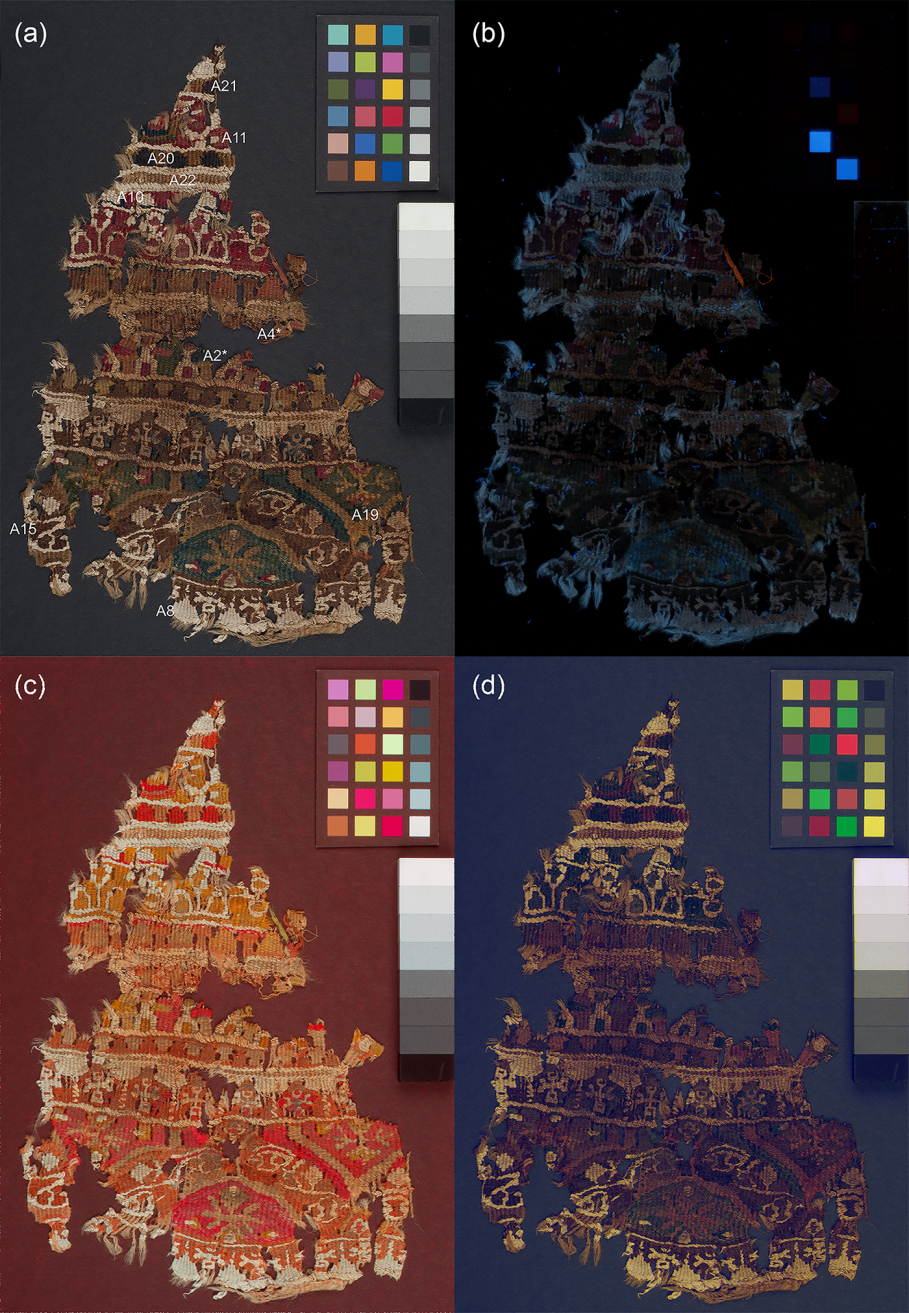
(a) Visible-reflected (VIS); (b) UV-induced visible luminescence (UVL); (c) Infrared-reflected false colour (IRRFC); (d) UV-reflected false colour (UVRFC) images of textile fragment A. The labelled areas are shown in Fig 7 at higher magnification.

**Fig 6:**
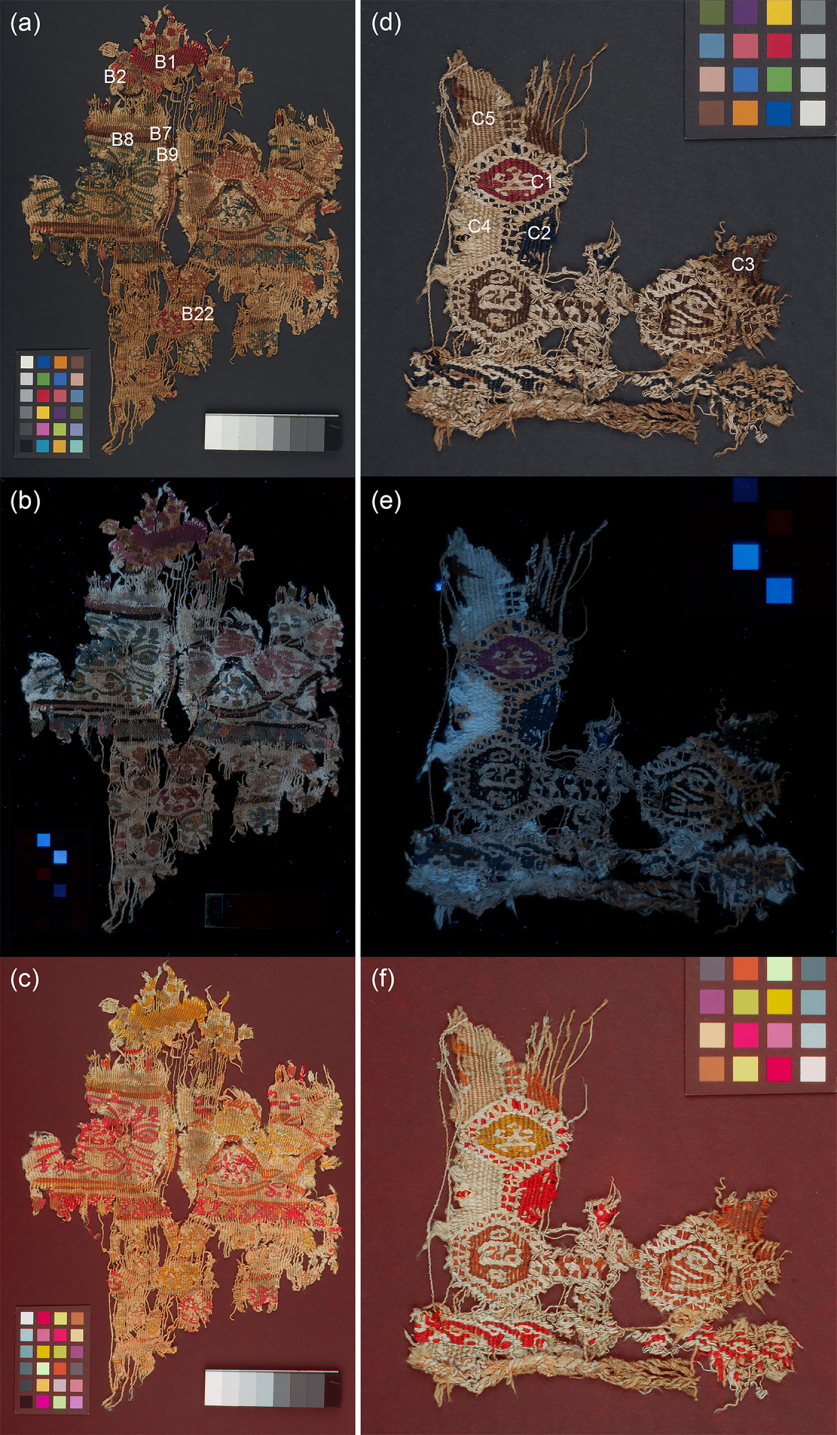
(a) Visible-reflected (VIS); (b) UV-induced visible luminescence (UVL) and (c) Infrared-reflected false colour (IRRFC) images of textile fragment B and (d), (e) and (f) the analogous images for textile fragment C. The labelled areas are shown in Fig 7 at higher magnification.

Fig 5 and Fig 6 show selected multispectral images of the furnishing textile fragments A—C from Wadi Sarga. Although initially these appear more complex and harder to interpret, systematic observation of the properties exhibited by the materials recorded in these images, as discussed in the following sections, yields important information.

#### UV-induced visible luminescence imaging (UVL)

**Fig 7:**
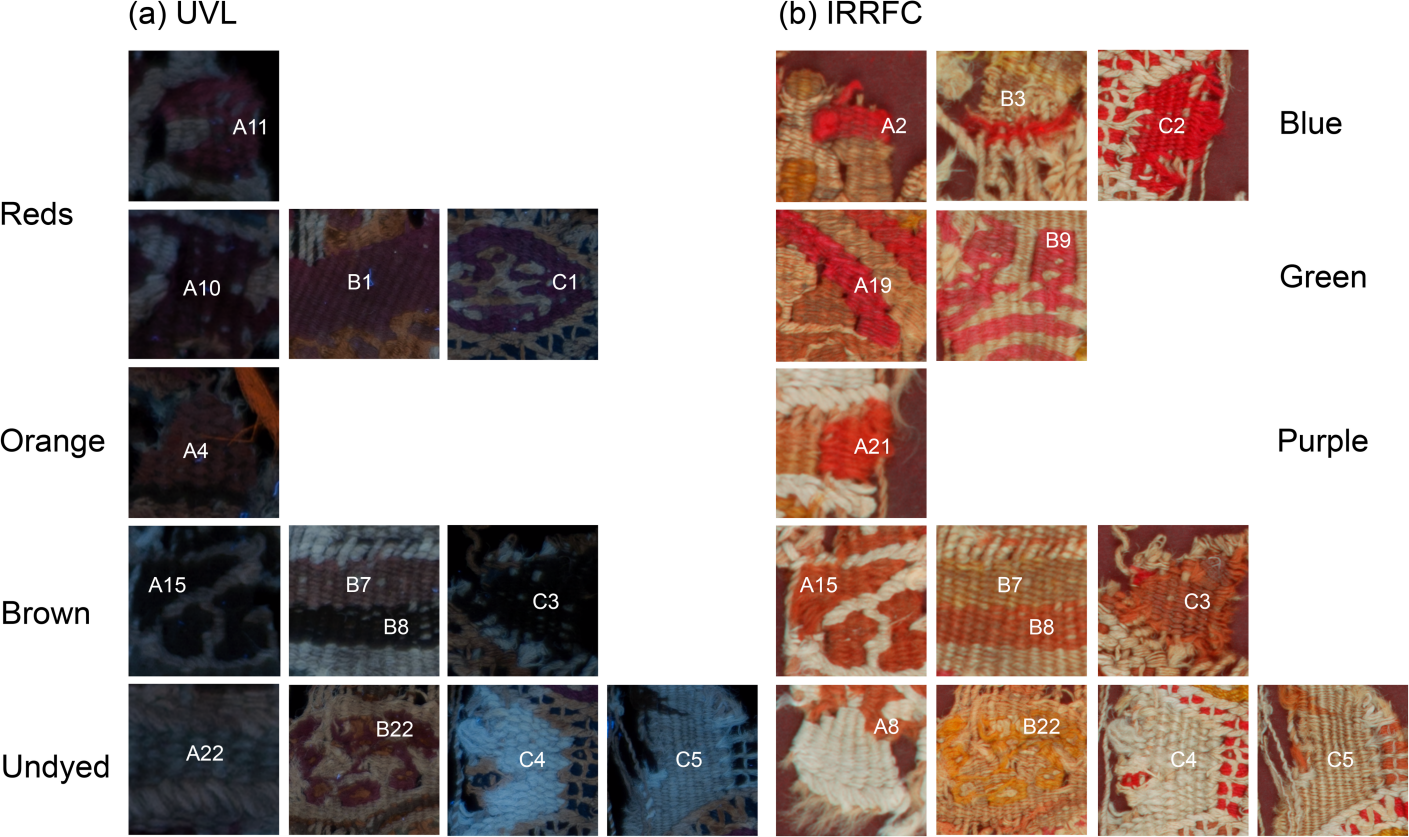
Details from the (a) UV-induced visible luminescence (UVL) and (b) Infrared-reflected false colour (IRRFC) images of textile fragments A–C.

The three textile fragments show differently luminescing areas of colourants (Fig 5B and Fig 6B and 6E) under UV light. Examples of these areas are shown in more detail in Fig 7A and are discussed below:

In textile A, the deeper reds seen in the visible images (for example, at A10), absorb UV and appear darker in the UVL images. Lighter-coloured reds or dark pinks (such as at A11) however, are observed to emit pink luminescence. In textile fragments B and C, the dark red areas (such as at B1 and C1) also appear quite dark or very weakly emitting in the UVL image. This is reminiscent of the behaviour observed for the dark red stripe in the child’s sock.

Areas which appear orange in the visible images of fragments A and B (see for example; A4 and B7) are also slightly luminescent in the UVL images. As previously noted, the luminescence from these areas may suggest the presence of a luminescent red colourant, such as madder, either alone or used in combination with another dye.

In all three textile fragments, areas which appear brown in the visible images (see for example; A15, B8 and C3) are very highly UV absorbing, appearing very dark in the UVL images, and may suggest the use of tannins [35].

Differences in the properties of the undyed yarns (see for example A8 vs. A22 or C4 vs. C5) are observed in all three textile fragments. In general, this may be related to their plant vs proteinaceous origins with wool and silk usually appearing bluish under UV, whereas lignocellulosic materials, such as cotton, linen or hemp, appear beige or slightly pink. Of particular note are the decorative “knots” observed in areas of fragment B (see for example B22), which, although similar in appearance to the background in the visible image, appear very different in the UVL image, emitting beige/pink luminescence.

An additional observation is that both the UVL images of fragments A and B show clumps of fibres, which display very intense orange luminescence and are clearly not original to these pieces, as the emission is too intense for a natural dye of this period. It is likely that these have become associated with the pieces in error.

#### Infrared-reflected imaging (IRR) and infrared-reflected false colour (IRRFC)

As previously, the materials in these furnishing textiles are largely transparent to IR, (see IRR images for textiles A—C in S1, S2 and S3 Figs, which appear very light), but here too the IRRFC images provide a map of indigo-containing areas, either alone or present in various mixtures (Fig 5C and Fig 6C and 6F). Examples of these areas are shown in more detail in Fig 7B. Thus, for fragments A and B, areas that appear very bright red are observed in the IRRFC images, which correlate to the areas of blue yarns in the textile fragments (see for example, A2 and B3), suggesting these are dyed chiefly with indigo. This is also the case for areas of textile C (primarily at C2), which are observed to be very bright red in the IRRFC image. In this case the IRRFC image is particularly successful in highlighting that, although these yarns appear almost black visually, they are in reality dark blue.

In addition, for both fragments A and B, various tones of salmon and bright orange are also observed in the IRRFC images, which can be associated with areas that appear green (such as A19 and B9) or purple in the visible images, respectively. Of particular note are the dark square shapes near the top of textile A (A20 and A21), which appear very dark brown in the visible image, but which are shown to contain indigo from the bright orange colour observed in the IRRFC image.

Duller orange areas can also be observed in the IRRFC images of all three textile fragments. These are associated in the visible images with areas of brown dyed yarns (see for example; A15, B8 and C3) and in the UVL images with areas of strong UV absorbance. The observation of this combined behaviour is in-keeping with the presence of tannins in these yarns and attests to the benefits of using both images for the purposes of interpretation.

Finally, the IRRFC images of all three textile fragments highlight the variations in the properties of the undyed yarns, confirming the observations from the UVL images that these are likely very different in origin. Of particular note are the stark differences between the reflectance properties of the different yarns in textile A, with the brighter yarns bordering areas of darker beige (such as at A22), and in textile C, where the contrast between the areas at C4 and C5 is particularly evident. The complementarity of the UVL and IRRFC images in the identification of these differences is important; for example, the distinctly emitting decorative “knots” observed in the UVL image of fragment B (B22), are not as evident in the IRRFC image.

#### UV-reflected imaging (UVR) and infrared-reflected false colour (UVRFC)

Under UV irradiation the materials which compose the textile fragments mostly absorb UV and, as such, the UVR images again appear very dark (see UVR images for textiles A—C in S4, S5 and S6 Figs).

However, of note are the differences in reflectance behaviour observed for the undyed yarns in the areas which appear bright in IRRFC and emit beige luminescence in the UVL images. These appear light coloured in the UVRFC image (Fig 5D) and are hence highly transparent in both the UV and visible regions. Treatments such as bleaching can lead to fibres that have a high degree of UV-Vis transparency [36], so this may be evidence that these fibres have been treated in some way.

#### Novel MSI developments–preliminary results

Two novel MSI methods, visible-induced visible luminescence imaging (VIVL) and multiband-reflected imaging (MBR), were applied to the study of two of the four textiles considered in this work: the child’s stripy sock and the furnishing textile fragment A.

#### Visible-induced visible luminescence imaging (VIVL)

**Fig 8:**
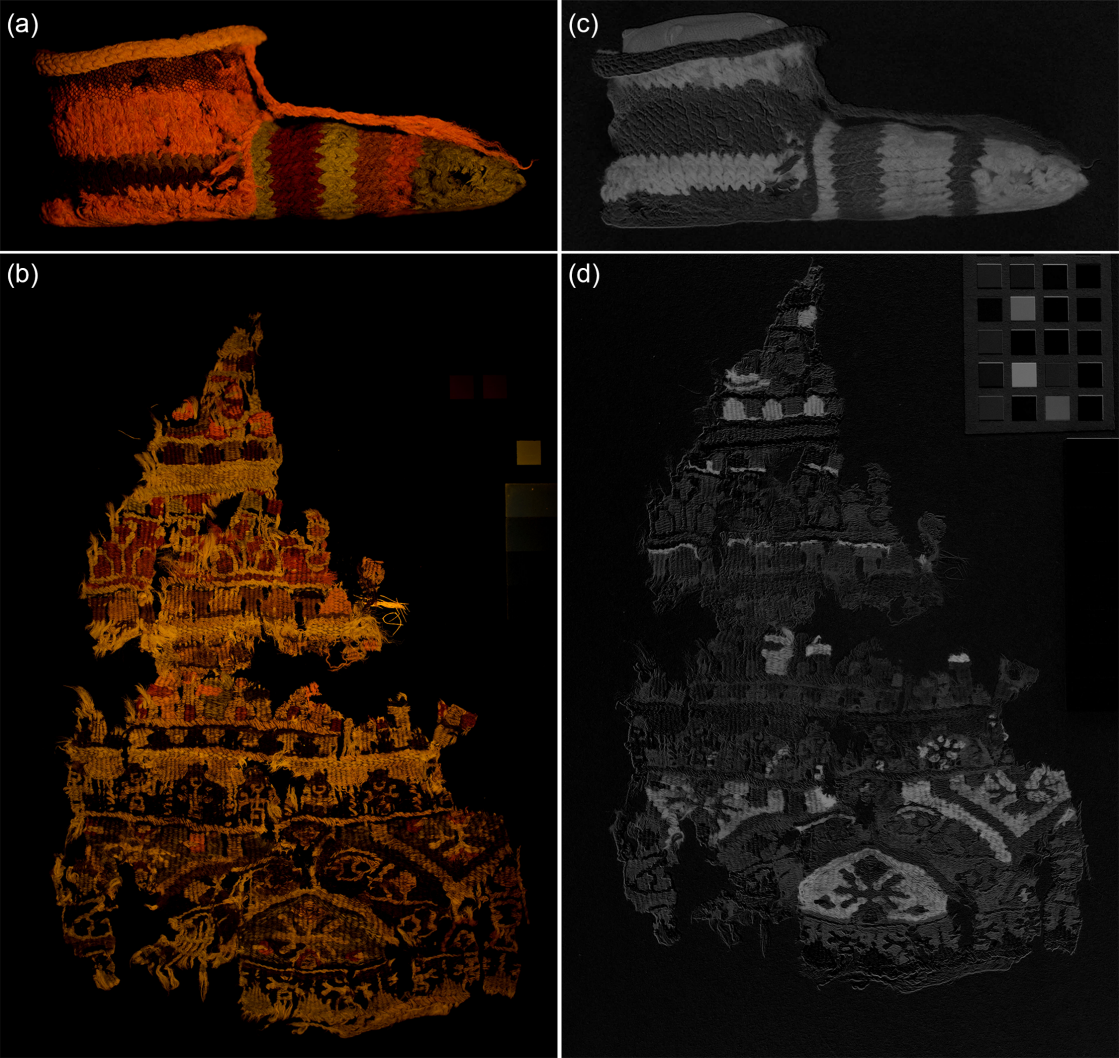
(a) and (b) Visible-induced visible luminescence (VIVL) and (c) and (d) multiband-reflected (MBR) images of (top) a child’s stripy sock and (bottom) textile fragment A.

Fig 8A shows the VIVL image acquired from the child’s sock. Comparison with the UVL image (Fig 4B) shows some key similarities and differences between these methods. In analogy with the UVL image, the areas of bright luminescence observed from stripes S3 and S6 dominate the image, but these appear more orange rather than bright pink. In addition, the variation in the luminescence properties for the green areas observed in the UVL images, where stripe S9 appeared much brighter and bluer than stripes S7 and S12, are no longer as apparent and a weak yellow-green luminescence from all the green stripes is observed in the VIVL image. These differences can be ascribed to the removal of the effects of background luminescence emitted by the fibres [11]. As discussed, the longer excitation wavelength used for the VIVL method avoids their absorption maximum, thus suppressing background luminescence, which in this case gave a blue cast to the luminescence being observed in the UVL images. As a result, the luminescence observed in the VIVL is more selective to the emission from the colourant rather than the combination of emissions from colourant and fibre.

In addition, as the VIVL method targets the collection of luminescence in the yellow-red region of the visible spectrum, where most dyes based on red colourants will emit if luminescent, areas containing red colourants are particularly highlighted in these images. This is particularly evident in the VIVL image of textile A in Fig 8B, where areas of red, and particularly lighter-coloured reds and orange in the top half of the textile fragment are particularly emphasised in comparison to the UVL image (Fig 5B).

Also noteworthy is how the bluish luminescence from fibres observed in the UVL image (from areas such as A22) is no longer apparent in the VIVL image due to the suppression of background luminescence resulting from the longer excitation wavelength used.

#### Multiband-reflected imaging (MBR)

Fig 8C and 8D show the MBR images acquired from the child’s sock and textile fragment A. As discussed, this method specifically targets the particular reflectance properties of colourants, in this case indigo, by acquiring reflectance images in multiple bands and subtracting these. The resulting grey-scale images produce a map of the indigo-containing areas with these appearing bright white in the final images.

This almost binary test for the presence of indigo, either alone or in mixtures, is visually arresting, as the indigo-containing areas are in stark contrast against the rest of the textile which appears dark. It is particularly powerful when the appearance of the blue colourant is not evident (for example, in areas which appear black) or when it is present in mixtures with other dyes. The green and purple coloured stripes of the child’s sock or the dark square shapes near the top of textile A (A20 and A21), which appear very dark brown in the visible image but appear very bright in the MBR image, are good examples of this.

However, unlike IRRFC images which can offer clues as to which colourant the indigo may be combined with, this method does not offer such subtleties. In addition, nuances such as the difference in yarn composition between stripes S4 and S5 are also lost. Nevertheless, when dealing with large or highly complex textiles with intricate designs, the straightforwardness afforded by the simple mapping of all areas containing indigo may be of more practical value than such level of detail.

### Summary of findings from MSI data and guidelines for interpretation

**Fig 9:**
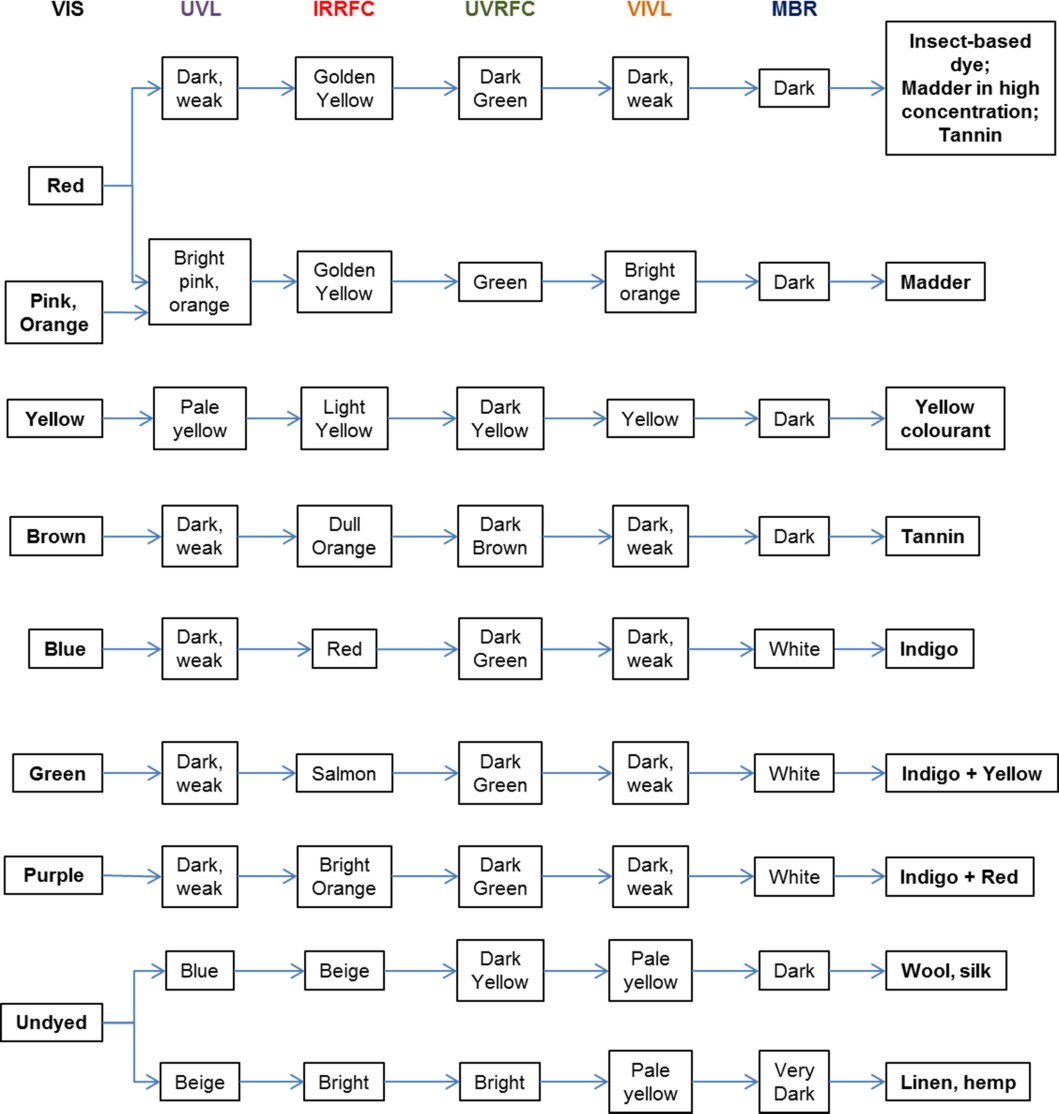
A flowchart summarising the main observations and interpretations made from the multispectral images collected in this investigation.

Based on the MSI results observed for the four objects under study, it was possible to summarise the main observations and interpretations made from the images collected in this investigation in the form of a chart (Fig 9).

One of the strengths of this approach is that it enables the observations made from the complete set of images acquired to be combined, providing a more rigorous description of the reflectance, absorbance and luminescence properties of the material being observed. It is this combination that is often powerfully diagnostic for the identification of specific colourants or fibre types.

The starting point is the property observed in VIS images, for example a colour or lack thereof. The flowchart then considers the behaviour observed for that property in each of the MSI image types and provides suggestions for the material or combination of materials which could be responsible for that behaviour. Although the complete set included in the flowchart is not required to reach some conclusions, the additional information provided by each technique enables a more refined suggestion to be made. Using this approach, it is possible to indicate the presence of colourants, such as indigo, madder and tannins, as well as their mixtures. These suggestions can then be probed further by applying the protocol described in Fig 3, as discussed in the following section.

### FORS, OM, HPLC-MS and SEM-EDX

The MSI observations were further investigated by FORS, OM, HPLC-MS and SEM-EDX according to the protocol described in Fig 3. Some details are reported in the following paragraphs and are summarised in Table 2 and Table 3.

**Table 2:** Summary of the analytical results for the samples taken from the sock and textile fragment A. Sample locations are detailed in Fig 1.

Sample	Microscopy	MSI	FORS	SEM-EDX	HPLC-MS	Technique
**Sock**						
S1_Yellow	Yellow fibres	Unidentified yellow colourant	Abs max at c. 420 nm; unidentified yellow colourant Abs feature between 500–600 nm; unidentified red colourant	Alum-mordanted	*Reseda luteola* (weld) *Rubia spp*. (madder)	Possibly double-dyed (yellow over red) yarn or fleece
S2_Purple	Dark red and blue fibres	Mixture of Indigo or woad and a red colourant	Abs max at 510 and 545 nm; a red dye of plant origin Abs max at 660 nm; indigo or woad	Red fibre: Alum-mordanted Blue fibre: Alum-mordanted (?)	*Rubia spp*. (madder) *Isatis tinctoria* (woad) or *Indigofera tinctoria* (indigo)	Yarns or fleeces of different colours (blue and red) twisted or spun together
S3_Orange/Red	Light red/orange fibres	Red colourant, likely madder	Abs max at 510 and 545 nm; a red dye of plant origin	Alum-mordanted	*Rubia spp*. (madder)	Yarn or fleece dyed with a single colour (red)
S4_Blue	Dark blue fibres	Indigo or woad	Abs max at 660 nm; indigo or woad	Alum-mordanted (?)	*Isatis tinctoria* (woad) or *Indigofera tinctoria* (indigo) *Rubia spp*. (madder)	Possibly double-dyed (blue over red) yarn or fleece
S5_Dark purple	Dark brown/purple and light brown fibres (faded)	Mixture of Indigo or woad and a red colourant (?)	Abs max at 510 and 545 nm; a red dye of plant origin Abs max at 660 nm; indigo or woad	Purple fibre: Alum-mordanted	*Rubia spp*. (madder) *Isatis tinctoria* (woad) or *Indigofera tinctoria* (indigo)	Possibly double-dyed (blue over red) yarn or fleece
S6_Orange/Red	x	Red colourant, likely madder	Abs max at 510 and 545 nm; a red dye of plant origin	x	x	x
S7_Dark Green	x	Mixture of Indigo or woad and a yellow colourant	Abs max at 660 nm; indigo or woad Abs max at c. 420 nm; unidentified yellow colourant	x	x	x
S8_Red	Bright red fibres	Red colourant; madder in high concentration, an insect-based dye or tannin- containing	Abs max at 510 and 545 nm; a red dye of plant origin	Alum-mordanted	*Rubia spp*. (madder)	Yarn or fleece dyed with a single colour (red), possibly with multiple dye baths
S9_Light Green	Light blue fibres	Mixture of Indigo or woad and a yellow colourant	Abs max at 660 nm; indigo or woad Abs max at c. 420 nm; unidentified yellow colourant	Unmordanted?	*Isatis tinctoria* (woad) or *Indigofera tinctoria* (indigo)	Yarn or fleece dyed with a single colour (blue), possible presence of faded yellow dye
S10_Purple	x	Mixture of Indigo or woad and a red colourant	Abs max at 510 and 545 nm; a red dye of plant origin Abs max at 660 nm; indigo or woad	x	x	x
S11_Orange/Red	x	Red colourant, likely madder	Abs max at 510 and 545 nm; a red dye of plant origin	x	x	x
S12_Dark green	Yellow and green fibres	Mixture of Indigo or woad and a yellow colourant	Abs max at 660 nm; indigo or woad Abs max at c. 420 nm; unidentified yellow colourant	Green fibre: Alum-mordanted Yellow fibre: Alum-mordanted	*Reseda luteola* (weld) *Isatis tinctoria* (woad) or *Indigofera tinctoria* (indigo)	Combination of yarn or fleece dyed with a single colour (yellow) and green double-dyed (yellow and blue) yarn or fleece
**Textile Fragment A**						
A1_Red	Bright red fibres	Red colourant; madder in high concentration, an insect-based dye or tannin- containing	Abs max at 510 and 545 nm; a red dye of plant origin	Alum-mordanted	*Rubia spp*. (madder)	Yarn or fleece dyed with a single colour, possibly with multiple dye baths
A2_Blue	Dark blue fibres	Indigo or woad	Abs max at 660 nm; indigo or woad	Unmordanted	*Isatis tinctoria* (woad) or *Indigofera tinctoria* (indigo)	Yarn or fleece dyed with a single colour (blue)
A3_Brown	Dark and light brown fibres?	Tannin-containing	No discernible spectral features	Unmordanted (?)	Tannins	Yarn or fleece dyed with a single colour, with evident fading
A4_Orange	Dark yellow/orange fibres	Red colourant, likely madder	Abs max at 510 and 545 nm; a red dye of plant origin Abs max at c. 420 nm; unidentified yellow colourant	Alum-mordanted	*Reseda luteola* (weld) *Rubia spp*. (madder)	Possibly double-dyed (yellow over red) yarn or fleece or
A5_Green	Dark green fibres	Mixture of Indigo or woad and a yellow colourant	Abs max at 660 nm; indigo or woad Abs max at c. 420 nm; unidentified yellow colourant	Alum-mordanted	*Reseda luteola* (weld) *Isatis tinctoria* (woad) or *Indigofera tinctoria* (indigo)	Double-dyed (yellow and blue) yarn or fleece
A6_Red (loose fibre)	Light red/orange fibres	Red colourant, likely madder	Abs max at 510 and 545 nm; a red dye of plant origin Abs max at c. 420 nm; unidentified yellow colourant	Alum-mordanted	*Rubia spp*. (madder) *Reseda luteola* (weld)	Yarn or fleece dyed with a mixture of colours (red and yellow)
A7_Yellow	Yellow fibres	Unidentified yellow colourant	Abs max at c. 420 nm; unidentified yellow colourant	Alum-mordanted	*Reseda luteola* (weld)	Yarn or fleece dyed with a single colour (yellow)
A8_White	White fibres	Linen or hemp	No discernible spectral features	Unmordanted	x	-
A9_Red	-	Red colourant; madder in high concentration, an insect-based dye or tannin- containing	Abs max at 510 and 545 nm; a red dye of plant origin	-	-	-
A10_Red	-	Red colourant; madder in high concentration, an insect-based dye or tannin- containing	Abs max at 510 and 545 nm; a red dye of plant origin	-	-	-
A11_light red	-	Red colourant, likely madder	Abs max at 510 and 545 nm; a red dye of plant origin Abs max at c. 420 nm; unidentified yellow colourant	-	-	-
A12_Light red	-	Red colourant, likely madder	Abs max at 510 and 545 nm; a red dye of plant origin Abs max at c. 420 nm; unidentified yellow colourant	-	-	-
A13_Orange	-	Red colourant, likely madder	Abs max at 510 and 545 nm; a red dye of plant origin Abs max at c. 420 nm; unidentified yellow colourant	-	-	-
A14_Orange	-	Red colourant, likely madder	Abs max at 510 and 545 nm; a red dye of plant origin Abs max at c. 420 nm; unidentified yellow colourant	-	-	-
A15_Brown	-	Tannin-containing	No discernible spectral features	-	-	-
A16_Brown	-	Tannin-containing	No discernible spectral features	-	-	-
A17_Blue	-	Indigo or woad	Abs max at 660 nm; indigo or woad	-	-	-
A18_Blue	-	Indigo or woad	Abs max at 660 nm; indigo or woad	-	-	-
A19_Green	-	Mixture of Indigo or woad and a yellow colourant	Abs max at 660 nm; indigo or woad Abs max at c. 420 nm; unidentified yellow colourant	-	-	-
A20_Purple/dark brown	-	Mixture of Indigo or woad and a red colourant	Abs max at 510 and 545 nm; a red dye of plant origin Abs max at 660 nm; indigo or woad	-	-	-
A21_Purple/dark brown	-	Mixture of Indigo or woad and a red colourant	Abs max at 510 and 545 nm; a red dye of plant origin Abs max at 660 nm; indigo or woad	-	-	-
A22_Undyed	-	Wool	No discernible spectral features			

**Table 3:** Summary of the analytical results for textile fragments B and C. Sample locations are detailed in Fig 2.

Sample	MSI	FORS
**Textile fragment B**		
B1_Deep red	Red colourant; madder in high concentration, an insect-based dye or tannin- containing	Abs max at 510 and 545 nm; a red dye of plant origin
B2_Light red	Red colourant, likely madder	Abs max at 510 and 545 nm; a red dye of plant origin Abs max at c. 420 nm; unidentified yellow colourant
B3_Dark blue	Indigo or woad	Abs max at 660 nm; indigo or woad
B4_Purple	Mixture of Indigo or woad and a red colourant	Abs max at 510 and 545 nm; a red dye of plant origin Abs max at 660 nm; indigo or woad
B5_Green	Mixture of Indigo or woad and a yellow colourant	Abs max at 660 nm; indigo or woad Abs max at c. 420 nm; unidentified yellow colourant
B6_Pink	Red colourant, likely madder	Abs max at 510 and 545 nm; a red dye of plant origin Abs max at c. 420 nm; unidentified yellow colourant
B7_Orange	Red colourant, likely madder	Abs max at 510 and 545 nm; a red dye of plant origin Abs max at c. 420 nm; unidentified yellow colourant
B8_Brown	Tannin-containing	No discernible spectral features
B9_Green	Mixture of Indigo or woad and a yellow colourant	Abs max at 660 nm; indigo or woad Abs max at c. 420 nm; unidentified yellow colourant
B10_Blue	Mixture of Indigo or woad and a yellow colourant	Abs max at 660 nm; indigo or woad
B11_Undyed	Wool	No discernible spectral features
B12_Blue	Indigo or woad	Abs max at 660 nm; indigo or woad
B13_Yellow	Unidentified yellow colourant	Abs max at c. 420 nm; unidentified yellow colourant
B14_Pink	Red colourant, likely madder	Abs max at 510 and 545 nm; a red dye of plant origin Abs max at c. 420 nm; unidentified yellow colourant
B15_Orange	Red colourant, likely madder	Abs max at 510 and 545 nm; a red dye of plant origin Abs max at c. 420 nm; unidentified yellow colourant
B16_Yellow	Unidentified yellow colourant	Abs max at c. 420 nm; unidentified yellow colourant
B17_Green	Mixture of Indigo or woad and a yellow colourant	Abs max at 660 nm; indigo or woad Abs max at c. 420 nm; unidentified yellow colourant
B18_Purple	Mixture of Indigo or woad and a red colourant	Abs max at 510 and 545 nm; a red dye of plant origin Abs max at 660 nm; indigo or woad
B19_Blue	Indigo or woad	Abs max at 660 nm; indigo or woad
B20_Yellow	Unidentified yellow colourant	Abs max at c. 420 nm; unidentified yellow colourant
B21_Pink	Red colourant, likely madder	Abs max at 510 and 545 nm; a red dye of plant origin Abs max at c. 420 nm; unidentified yellow colourant
B22_Knot	Linen, hemp	No discernible spectral features
**Textile fragment C**		
C1_Deep red	Red colourant; madder in high concentration, an insect-based dye or tannin- containing	Abs max at 510 and 545 nm; a red dye of plant origin
C2_Dark blue	Indigo or woad	Abs max at 660 nm; indigo or woad
C3_Brown	Tannin-containing	No discernible spectral features
C4_Undyed Beige	Wool	No discernible spectral features
C5_Undyed beige	Wool	No discernible spectral features
C6_Undyed cream	Linen, hemp	No discernible spectral features
C7_Undyed cream	Linen, hemp	No discernible spectral features

#### Child’s stripy sock

**Fig 10:**
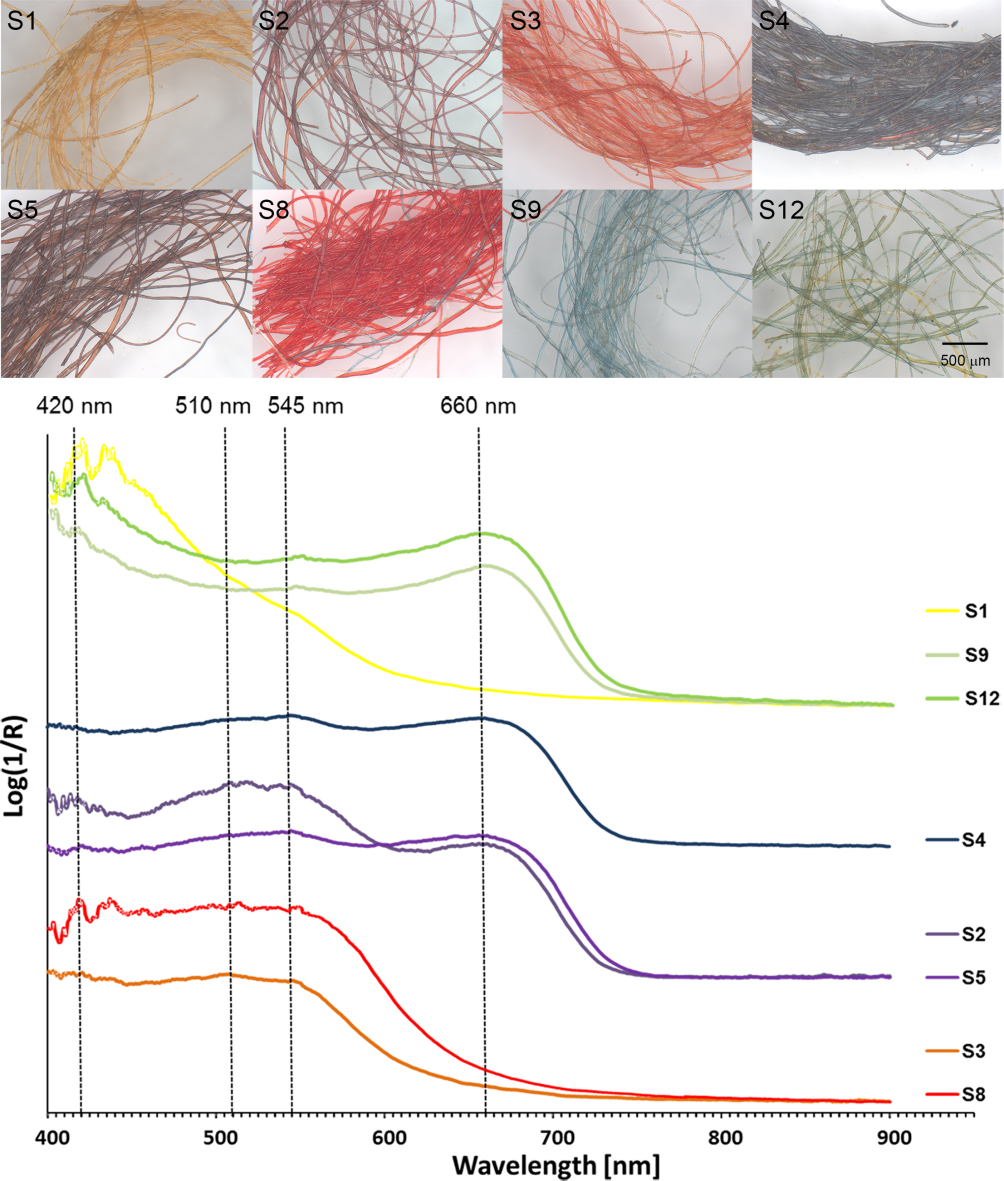
Micrographs of selected samples at 80x magnification and apparent absorption data obtained from FORS measurements of these areas.

The orange stripes, S3 and S6 were observed to emit bright pink luminescence in the UVL image (Fig 4B), which was interpreted as being associated with a red colourant, such as madder [32,33]. Fig 10 shows the apparent absorption data obtained from FORS measurements of these areas, which gave a pair of absorption maxima at 510 and 545 nm, indicative of a red dye of plant origin [14,15]. HPLC-MS analysis performed on a sample taken from stripe S3 showed the presence of alizarin, purpurin, pseudopurpurin, xanthopurpurin, rubiadin, munjistin and other anthraquinones associated with madder derived from a *Rubia* species (Table 2). *Rubia tinctorum* is the most likely source considering the geographical area, but the chemical distinction of the Rubiaceae plants from archaeological samples is never straightforward, due to the high number of factors that can affect the final dyestuff composition observed, such as the natural variability in the chemical composition of the madder root, the extraction, the dye preparation procedures and the analytical protocol adopted [37–44]. No evidence for components from a yellow dye was observed.

Surprisingly, the sample from deeper red stripe S8, which appeared notably darker in the UVL image, gave very similar results to sample S3 when analysed by both FORS and HPLC-MS (Table 2). Comparison of the fibres using OM (Fig 10) showed that the colourant in the stripe S8 is much brighter and more intense, perhaps suggesting several dye baths have been applied to the yarn or perhaps differences in pH. Differences in mordanting were discounted from the distribution of elements in the EDX analysis, which showed that, apart from a slightly elevated concentration of sodium and chlorine on the fibres of sample S3, both appear to be alum-mordanted. The differences in the luminescence properties thus appear to be associated to the amount of dye on the fibres rather than how this was applied.

In the case of the three green areas, the apparent absorption data obtained from FORS measurements (Fig 10) showed absorption maxima at 660 nm, characteristic of the presence of indigo, with some contribution in the area around 400 nm (absorption maximum at c. 420 nm), suggesting mixtures with yellow colorants. However, observation of the fibres using OM (Fig 10) highlighted differences in the samples of yarn taken from stripe S9 versus stripe S12, with the former appearing very lightly dyed or faded and seemingly composed only of blue fibres, and the latter a mixture of yellow and green fibres. HPLC-MS analysis also suggested that no components derived from yellow colourants were present in sample S9. However, since the presence of some yellow is suggested from both the FORS and MSI data, as well as visually, it is possible that these have faded or degraded to a degree which is below the limit of detection for this method. Isatin, indigotin and indirubin, which are the components consistent with woad (*Isatis tinctoria*) or *Indigofera* species were detected in sample S9, but only isatin and indigotin were detected in sample S12, highlighting another difference between the two samples. In addition, sample S12, containing green and yellow fibres, also showed the presence of luteolin, apigenin, chrysoeriol and several of their glycosides, which are typically associated with the yellow colorant weld (*Reseda luteola*). Two additional molecules with a pseudo-molecular ion at *m/z* 313 were also detected in all the samples containing this type of dye. These molecules have been recently identified in another work [45] and may be markers of a different variety of weld or dyeing process used. Although the green and yellow fibres were not analysed separately, this suggests the use of a combination of two differently dyed yarns twisted together, one of which was double-dyed using yellow and blue colourants to obtain green.

Differences in the luminescence properties were also observed between the dark purple stripe S5, and the visually similar stripes S2 and S10. FORS measurements (Fig 10) showed absorption maxima at 660 nm, together with a pair of absorption maxima at 510 and 545 nm, at each of these areas, suggesting a mixture of blue and red plant-based colourants. This mixture of colourants was also suggested by the bright orange colour observed in these areas in the IRRFC image (Fig 4D). This was confirmed by HPLC-MS analysis, which showed the presence of anthraquinones associated with madder derived from a *Rubia* species, as well as isatin and indigotin consistent with woad or an *indigofera* species, in both samples S2 and S5. In the sample from stripe S5, that appeared largely dark in the UVL image, the relative concentration of components from *Rubia* appears to be much higher than in sample S2, based on the relative abundance of chromatographic peak areas. A higher concentration of the dye may explain these properties in analogy with that observed for the deep red stripe (sample S8). However, the FORS data showed a weak apparent absorption associated with a plant-derived red dye for this area, and dark brown/purple and light brown fibres were observed using OM in this sample (Fig 10). By contrast, blue and lightly dyed red fibres were observed in sample S2, representative of the areas where weak pink luminescence was observed in the UVL image. Both were shown by EDX analyses to be similarly mordanted with alum. A possible explanation for the differences may lie in the technique used to dye the yarns: it is suggested that the dark brown/purple fibres are dyed sequentially (double-dyed), first with madder, perhaps at high concentration or several dye baths, responsible for the UV-absorbing effects, and then with indigo. The reflectance properties of the red colourant would mostly be obscured by the coating of indigo on the surface, partially explaining the results. A similar effect, where madder signals in the FORS spectra were masked due to a double-dyeing process with lac over madder, has been previously observed [14].

Some evidence for a yellow colourant with an apparent absorbance maximum at c. 420 nm was observed in the FORS measurements of the yellow stripe S1 (Fig 10). A slight feature was also observed at 500–600 nm. No evidence for a mixture of differently dyed yarns was observed from OM, however, HPLC-MS analysis revealed that a mixture of madder and weld was used to dye these yarns. The relative abundance of the anthraquinones from madder was very low compared to the flavonoids from weld, partially explaining the fact that no characteristic pink luminescence to suggest the presence of madder was observed in this area of UVL image. A similar result was obtained for the blue stripe S4. FORS indicated an absorption maximum at 660 nm, confirming the presence of indigo, and only blue fibres were observed with OM (Fig 10). However, HPLC-MS analysis highlighted the presence of the indigo markers together with all the markers of madder (*Rubia* sp.) with low relative abundances. In these cases, a double-dyeing technique may also explain these observations by causing a “hiding effect” of the underlying dye, but some cross-contamination from adjacent fibres cannot be excluded.

#### Textile fragment A

**Fig 11:**
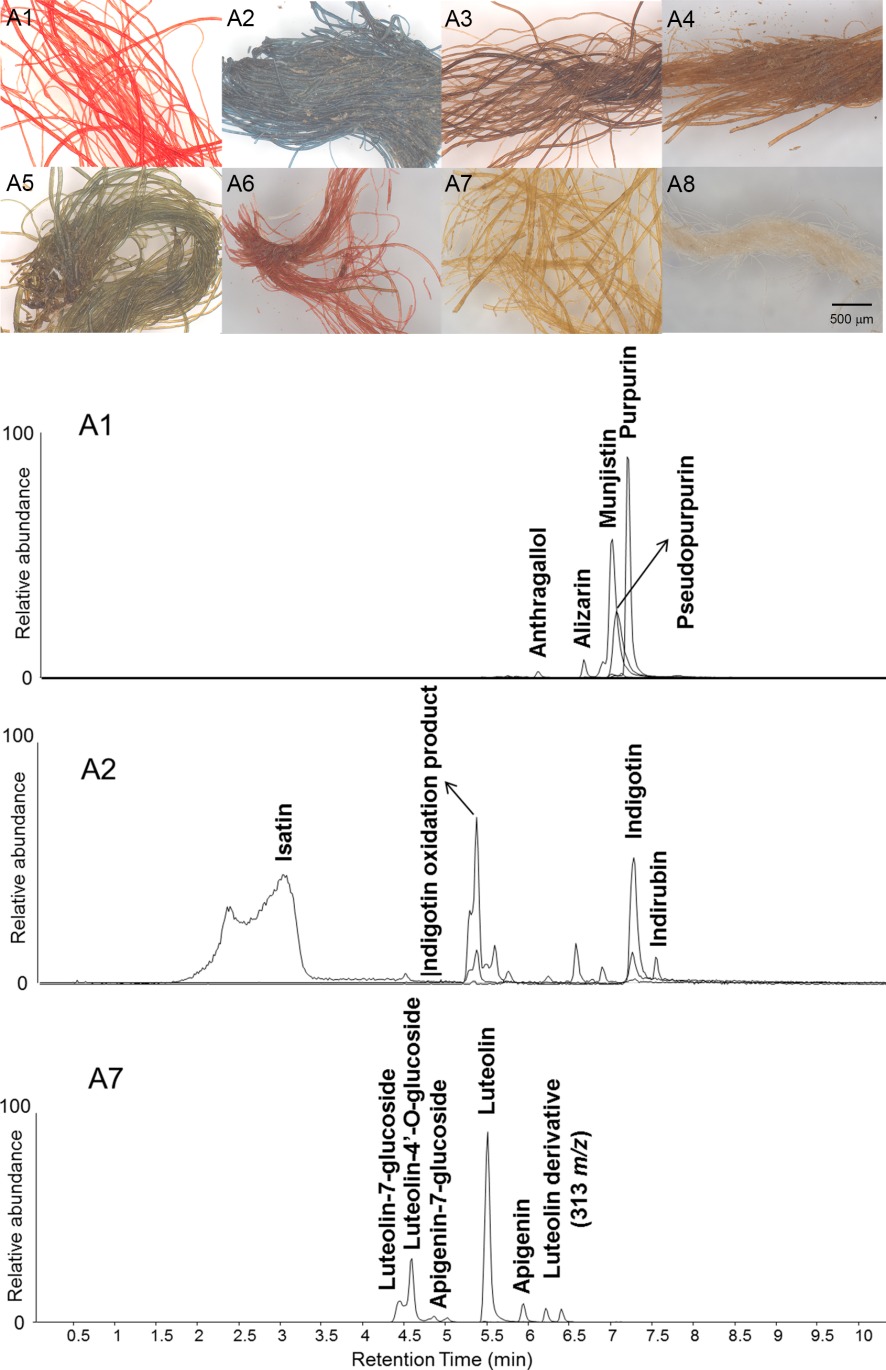
Micrographs of selected samples at 80x magnification and Extract Ion Chromatograms (EICs) obtained by HPLC-ESI-Q-ToF analysis of samples A1, A2 and A7, showing the dye compounds identified.

Differently luminescing areas of red colourants (Fig 5B), with the deeper reds (such as those at A1, A9 and A10) appearing darker, and the lighter-coloured reds (such as those at A11) emitting pink luminescence, were also observed in the UVL images of textile fragment A. The apparent absorption data obtained from FORS measurements of all these areas (Table 2) showed a pair of absorption maxima at 510 and 545 nm, indicative of a red dye of plant origin [14,15]. In the lighter red areas some contribution in the area around 400 nm (absorption maximum at c. 420 nm), suggesting possible mixtures with yellow colourants was observed. HPLC-MS analysis showed the presence of alizarin, purpurin, pseudopurpurin, xanthopurpurin, rubiadin and munjistin and other anthraquinones associated with madder derived from a *Rubia* species (Table 2) in both samples A1 and A6, taken from dark and light areas respectively. In addition, evidence for luteolin and several of its glycosides, associated with weld, was observed in sample A6, confirming the observations made from the FORS data. As previously, investigation of the fibres using OM (Fig 11) showed that the colourant in the darker red stripe (A1) is much brighter and more intense, thus the differences in the luminescence properties can again be ascribed to the dye concentration on the fibres.

Orange areas were also observed to be slightly luminescent in the UVL images, (Fig 5B). FORS measurements of selected areas (such as those at A4, A21 and A22; Table 2) showed similar results to those obtained for the lighter red coloured areas. HPLC-MS analysis of sample A4, however, showed that the proportion of red to yellow colourants appears more equivalent in these yarns. This is reflected from OM of this sample (Fig 11), which shows much yellower coloured fibres. However, from the data acquired it is not possible to tell whether this was obtained via a double-dyeing method or mixture of dyes in one dye vat.

Highly absorbing areas in the UVL image (Fig 5B), which appear brown in the visible image (A3, A17 and A18), were interpreted as suggestive of the use of tannins. Although iron(III)-tannin complexes have somewhat characteristically shaped FORS spectra [35,46], with a typical slow rise in the red region, no discernible spectral features, such as maxima, minima or inflection points are present [13]. As a result, the FORS measurements of these areas confirm little except for their high degree of infrared transparency, as evidenced from the IRRFC images, where these areas appear a dull orange colour. However, HPLC analysis (sample A3) revealed the presence of 4-hydroxybenzoic and ellagic acids, confirming the use of tannins in the dyeing of these yarns.

FORS measurements of the areas identified from the IRRFC image as containing indigo, either alone (as for examples at A2, A13 and A15) or present in various mixtures, all showed the characteristic apparent absorption maxima at 660 nm, confirming the presence of indigo (Table 2). In addition, the areas interpreted to be mixtures of indigo with reds (for example A14 and A16) also showed a pair of absorption maxima at 510 and 545 nm, indicative of a red dye of plant origin, whereas in the areas assigned as mixtures of indigo with yellow colourants (A2 and A23), an apparent absorbance maximum at c. 420 nm was observed. The purple yarns were not analysed by HPLC as these areas could not be sampled, however HPLC analysis confirmed both the presence of components characteristic of woad or and *indigofera* species in samples A2 and A5, as well as luteolin and luteolin-derived compounds in sample A5. Investigation of the fibres using OM (Fig 11) showed that the yarns from this green area were likely double-dyed.

The differences in the properties of the undyed yarns (see for example A8 vs. A22) noted from MSI were not clear from FORS measurements which showed only slight differences between these areas, but no clear spectral features. SEM analyses of a sample taken at A8 confirmed the fibres to be linen. A high level of degradation (multiple tangential and horizontal cracks in the fibres) and a high level of sodium compared to all the other samples may point towards a bleaching process performed on these fibres in order to obtain a white colour. This is in-keeping with observations made from the UVRFC images.

#### Textile fragments B and C

For these textiles only FORS was used in addition to the MSI images to investigate the nature of the colourants present. However, despite the absence of HPLC-MS data, it was possible to make some assignments based on the results obtained for the sock and textile fragment A.

The FORS results obtained (Table 3) were generally very similar to those observed for textile fragment A, therefore the use of similar colourants can be suggested. In particular, the difference in luminescence between dark red areas (B1 and C1) and lighter red areas (B2) was not translated into a difference in the FORS spectra, which indicated the presence of a plant-based red colourant, probably madder (absorption maxima at 510 and 545 nm) in both. Orange areas (B7 and B15) were also slightly luminescent in the UVL images and a mixture of madder and yellow was present. Brown areas (B8 and C3) revealed high absorption properties in the UVL images and appeared dull orange in IRRFC images, suggesting the presence of tannins.

Areas identified from the IRRFC image as containing indigo, either alone (B3, B10, B12, B19 and C2) or present in mixtures with yellow colourants (B5, B9 and B21) and red colourants (B4 and B18) all showed the characteristic apparent absorption maxima at 660 nm, confirming the presence of indigo. Absorption maxima at c. 400 nm were also generally observed for the green areas and absorption maxima at 510 and 545 nm (madder) were observed in the purple areas.

Only slight differences were observed between the areas of undyed yarns which appear bluish (C4 and C5) and beige (C6 and C7) under UV, however, no clear spectral features that could be diagnostic were observed. Previous studies however, did establish these to be wool and flax, respectively [47].

## Conclusions

This work has demonstrated the potential of MSI techniques to investigate the colourants and other characteristics of textile materiality non-invasively, by both adapting existing methods and developing new techniques specifically for the study of textiles. The need to follow scientifically-established protocols to ensure the reproducibility and comparability of results has been highlighted. Guidelines have been developed that aid in the systematic interpretation of the images, by reliably considering the combined photophysical properties being observed.

The four Late Antique textiles selected from the British Museum collection proved to be excellent case studies with which to test MSI techniques because, despite the colours appearing to be highly varied, they were obtained using a rather limited dye palette. Four main dye sources were identified, namely madder (*Rubia* sp.), weld (*Reseda luteola*), indigo (*Isatis* or *Indigofera tinctoria*) and tannins. Dyes from these sources, either alone or in mixtures, were identified by combining the information obtained from MSI, according to the guidelines proposed. Differences and similarities between and within the coloured areas of the various textiles were also established and confirmed by the FORS measurements.

However, some limitations were observed regarding the use of this completely non-invasive approach combining MSI and FORS.

The concentration of the dye appeared to play a role in the luminescence properties of dark red areas, which appeared to absorb or only weakly luminesce, thus complicating the interpretation of UVL images and possibly leading to the misidentification of such areas as perhaps containing insect-derived reds instead of madder.

The effects of background luminescence produced by the fibres can cause distracting and colour-modifying effects in lightly dyed or faded yarns. These effects were somewhat overcome by using VIVL methods, where the longer excitation wavelength used avoids the absorption maximum of most fibres, suppressing this background luminescence.

Moreover, yellow dyes could not be easily differentiated by FORS, as the various sources relevant to this context have very similar spectra. There was similarly a lack of diagnostic spectral characteristics for tannins and the different sources of undyed yarns.

Finally, the investigation of double-dyed yarns appeared particularly challenging to both MSI techniques and FORS measurements, as the information produced by the underlying dye can be hidden from these surface-responsive techniques.

These limitations have been overcome by integrating the use of MSI and FORS into a multi-analytical protocol. These techniques were in fact an important aid in planning a targeted and effective sampling strategy, narrowing down the number of additional measurements necessary, and facilitating comparisons between objects. OM, SEM-EDX and HPLC-MS were then applied to these samples, providing conclusive information on the dyeing technique, the mordanting and the botanical source of the colourants, respectively.

Future investigations to correlate the relative abundances of dye molecules observed in the chromatograms with the dye technique used to obtain mixed colours (double-dyed yarns and yarns composed of different colours that are dyed separately and twisted together) would be desirable.

The application of the full investigative protocol provided an increased knowledge of the materials and techniques used to create these pieces. Although the dye sources were in-keeping with that expected for the period and geographical location, these were used in ways which are testament to the subtleties of the dyers’ craft in Late Antiquity; they were able to produce an entire palette of colours using combinations of just three natural colourants; indigo, weld and madder.

Finally, the protocol described would undoubtedly be useful for the investigation of textiles in museum and historic collections more generally and future investigations are intended that will challenge the MSI approach with more complex dye palettes, such as those encountered in Asian or South American textiles. Since dyes respond differently to environmental factors, especially light, their identification is also useful for determining which textiles can be displayed safely.

## Supporting information

S1 Fig Infrared-reflected (IRR) image of textile fragment A. (TIF)

S2 Fig Infrared-reflected (IRR) image of textile fragment B. (TIF)

S3 Fig Infrared-reflected (IRR) image of textile fragment C. (TIF)

S4 Fig Ultraviolet-reflected (UVR) image of textile fragment A. (TIF)

S5 Fig Ultraviolet-reflected (UVR) image of textile fragment B. (TIF)

S6 Fig Ultraviolet-reflected (UVR) image of textile fragment C. (TIF)
