# Supplementary figures and images for: A multispectral imaging approach integrated into the study of Late Antique textiles from Egypt
Source: PLoS One. 2018 Oct 4;13(10):e0204699. doi: 10.1371/journal.pone.0204699 (PMC6171845; doi:10.1371/journal.pone.0204699)

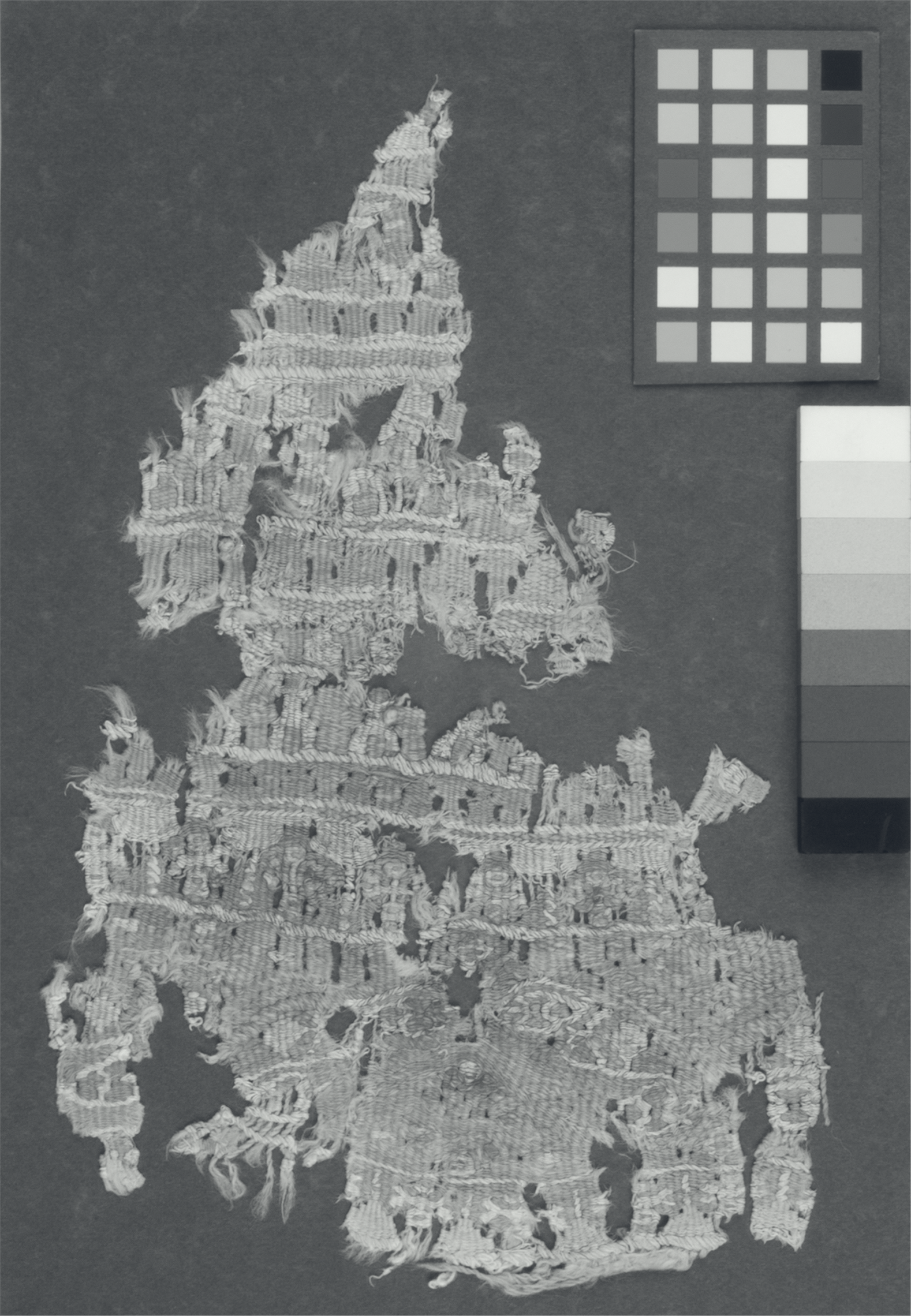

Supplement: S1 Fig — (TIF) [file pone.0204699.s001.tif]

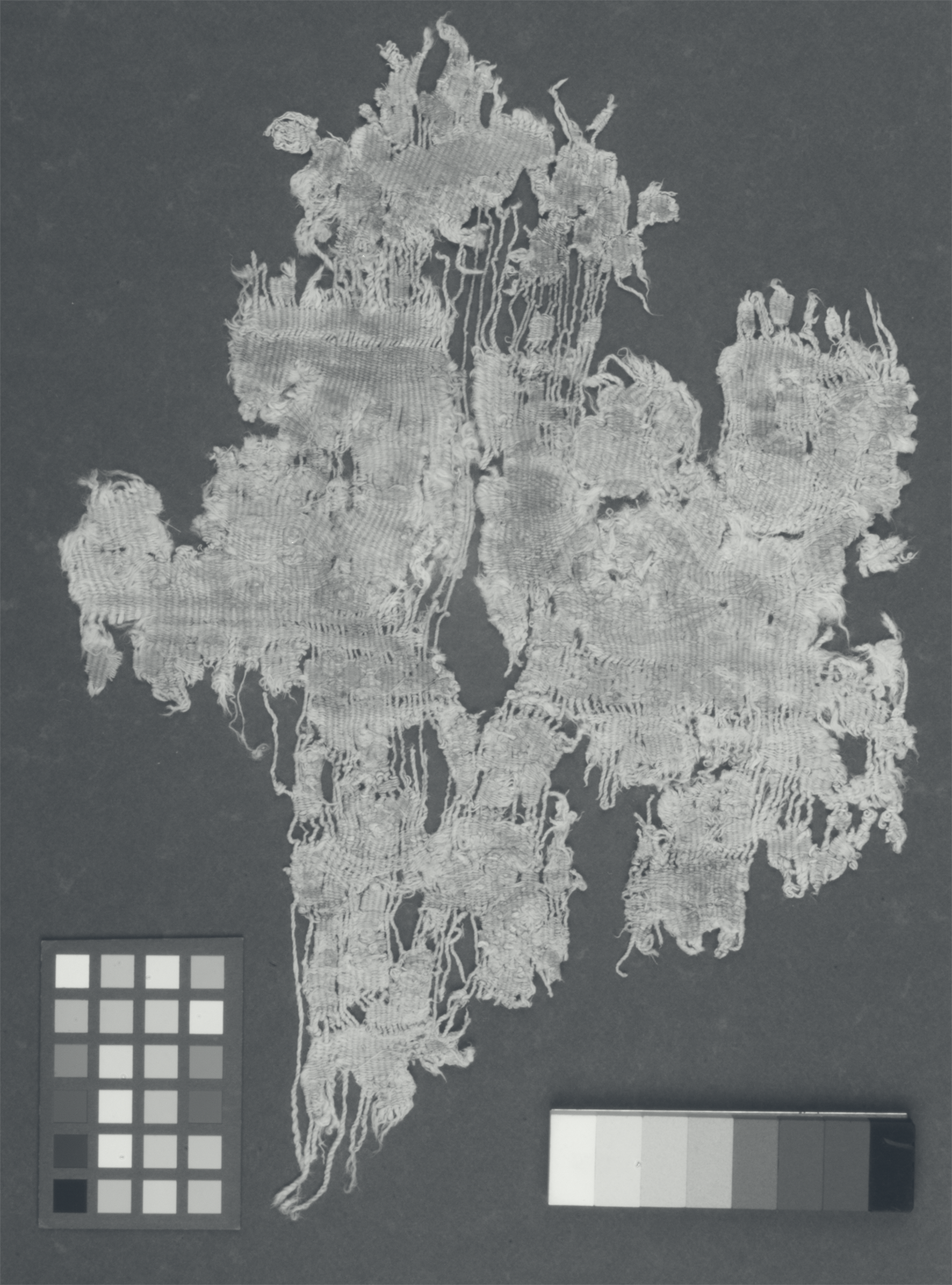

Supplement: S2 Fig — (TIF) [file pone.0204699.s002.tif]

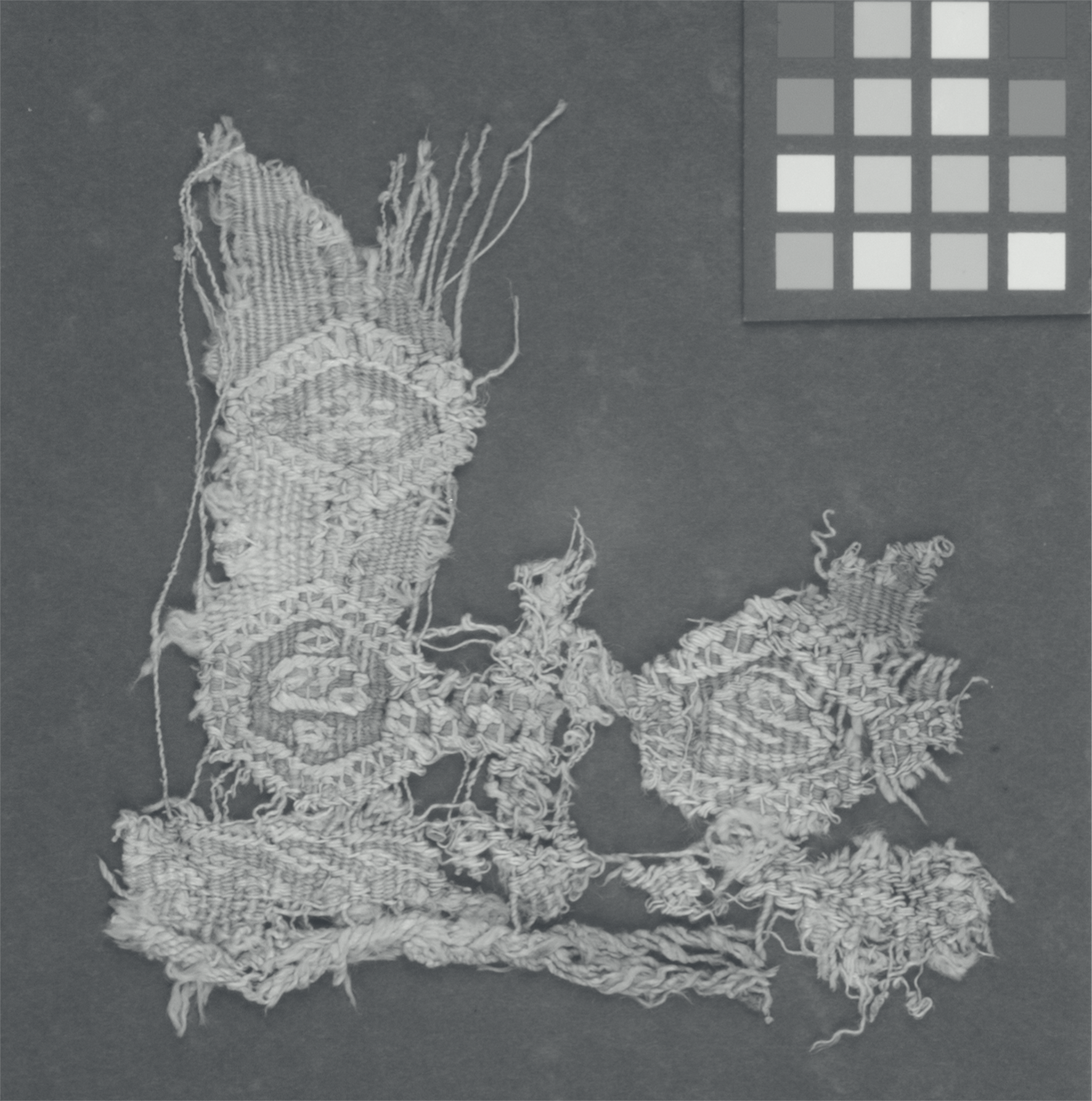

Supplement: S3 Fig — (TIF) [file pone.0204699.s003.tif]

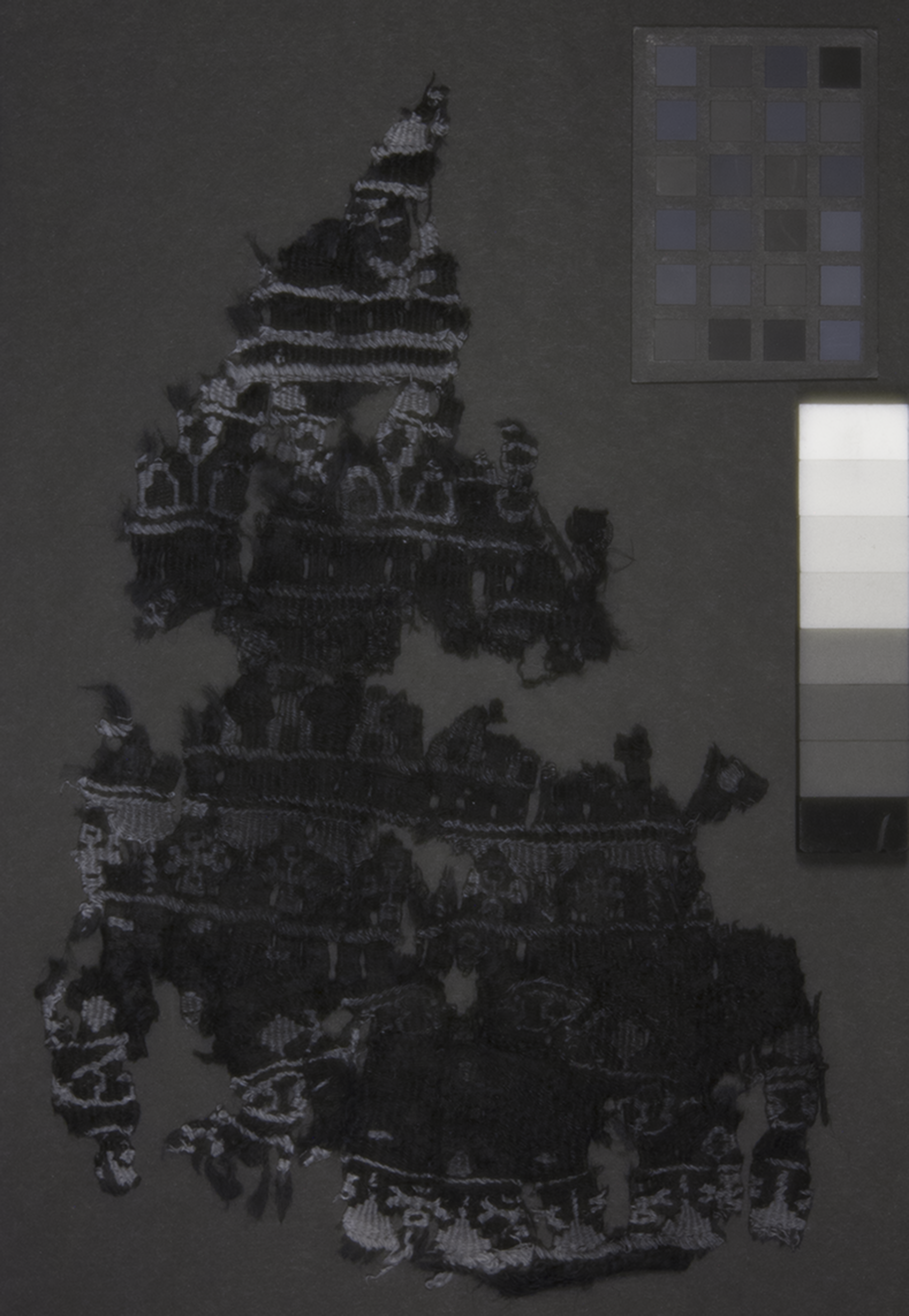

Supplement: S4 Fig — (TIF) [file pone.0204699.s004.tif]

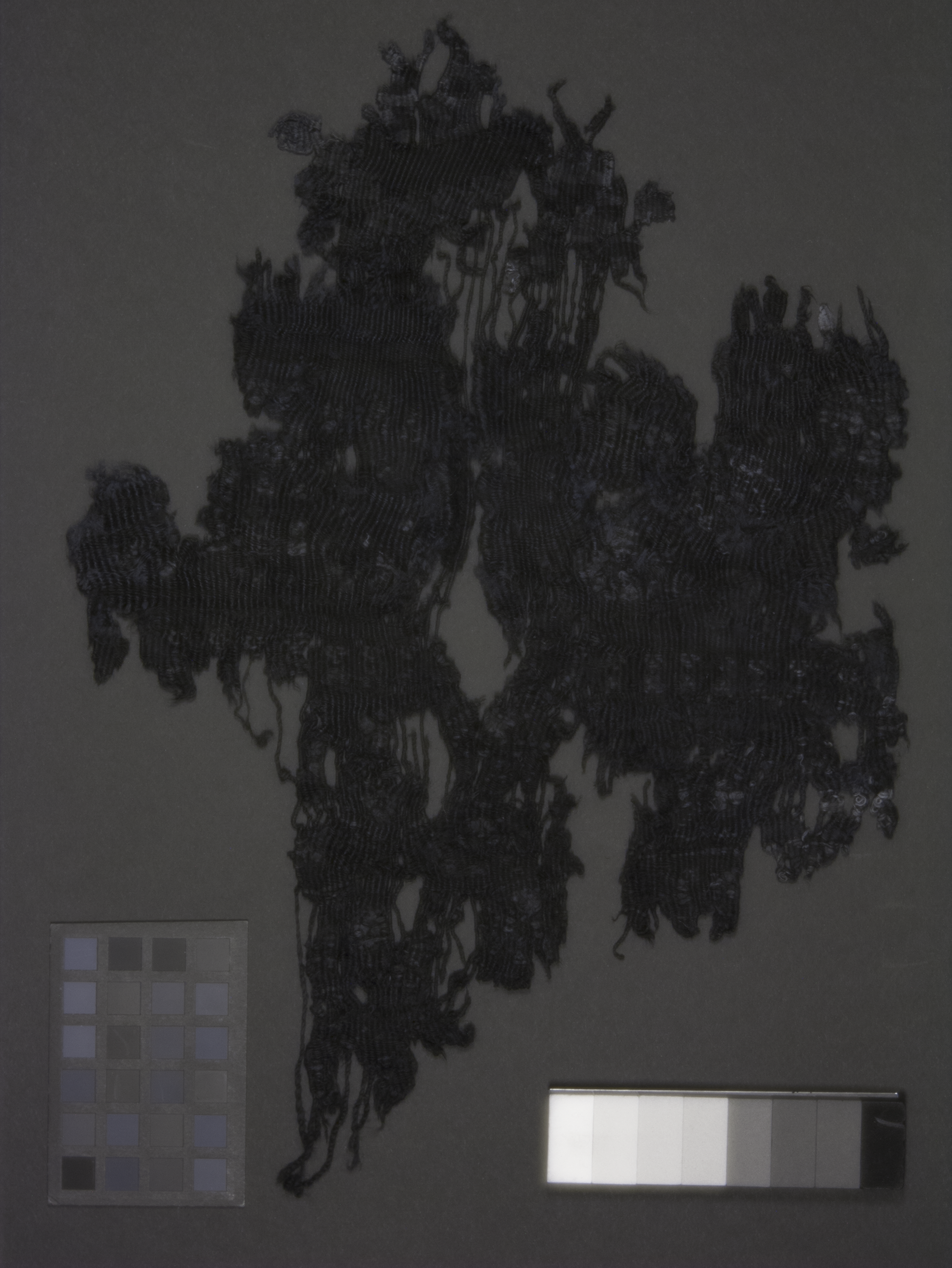

Supplement: S5 Fig — (TIF) [file pone.0204699.s005.tif]

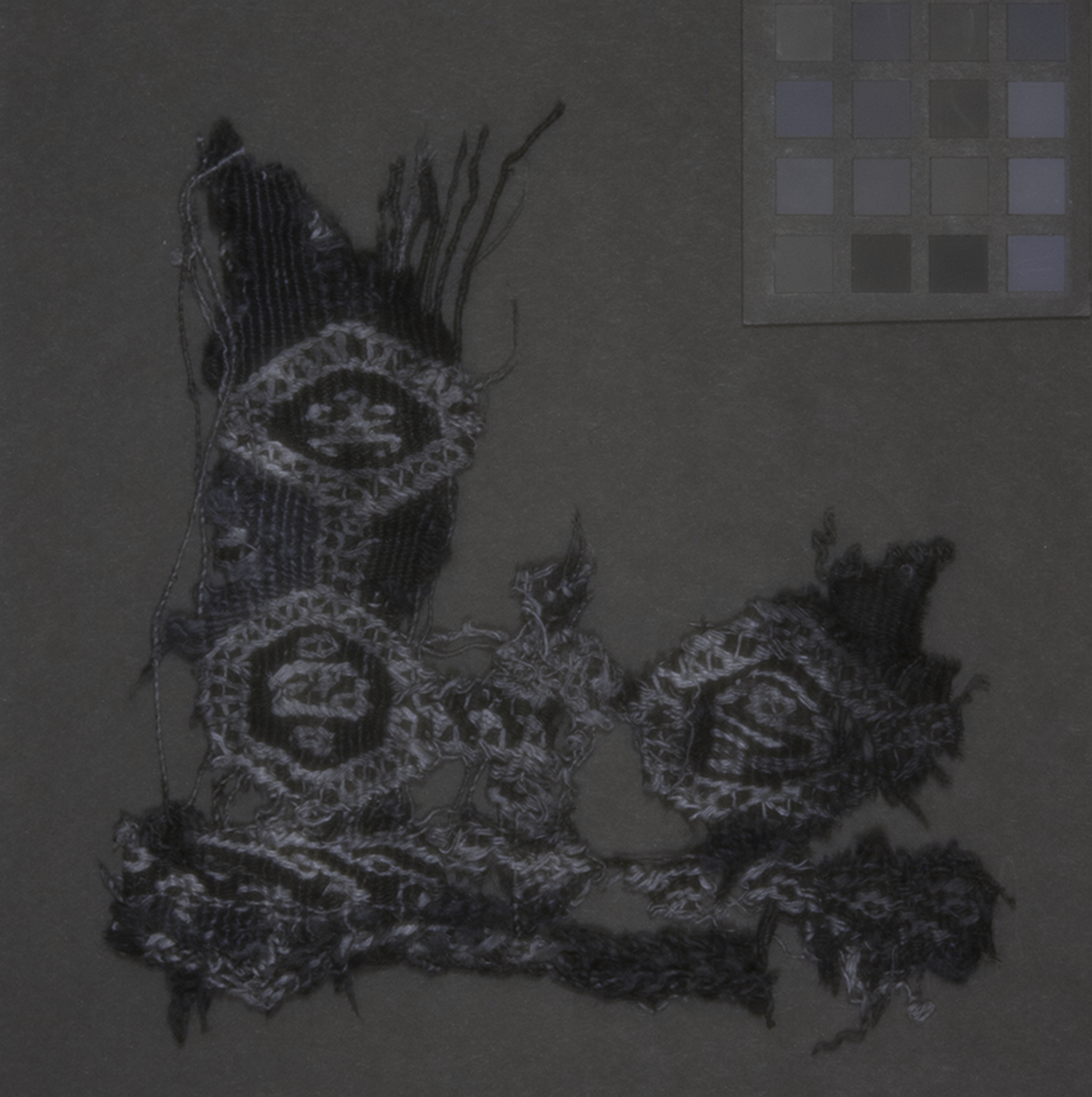

Supplement: S6 Fig — (TIF) [file pone.0204699.s006.tif]
